# Untargeted metabolomics by high resolution mass spectrometry coupled to normal and reversed phase liquid chromatography as a tool to study the in vitro biotransformation of new psychoactive substances

**DOI:** 10.1038/s41598-019-39235-w

**Published:** 2019-02-26

**Authors:** Sascha K. Manier, Andreas Keller, Jan Schäper, Markus R. Meyer

**Affiliations:** 1 Department of Experimental and Clinical Toxicology, Institute of Experimental and Clinical Pharmacology and Toxicology, Saarland University, Center for Molecular Signaling (PZMS), 66421 Homburg, Germany; 2 Chair of Clinical Bioinformatics, Saarland University, Saarbruecken, Germany; 3 State Bureau of Criminal Investigation Bavaria, München, Germany

## Abstract

In 2016, several synthetic cathinones were seized by the State Bureau of Criminal Investigation Bavaria in Germany. Due to their previous appearances in other countries their metabolism was already investigated in human urine as well as different *in vitro* models. These investigations were conducted using ordinary metabolism studies for drugs of abuse by using general knowledge about drug metabolism and visual comparison of mass spectra. The present study aimed to use untargeted metabolomics to support and improve those methods that highly depend on the investigators experience. Incubations were conducted using pooled human liver microsomes (pHLM) and the two cathinones 1-phenyl-2-(1-pyrrolidinyl)-1-butanone and 1-phenyl-2-(1-pyrrolidinyl)-1-heptanone. Samples were analyzed by LC-HRMS/MS using a metabolomics workflow consisting of a reversed phase or normal phase separation followed by electrospray ionization and full scan in positive or negative mode. LC-MS data was afterwards statistically evaluated using principal component analysis, t-distributed stochastic neighborhood embedding, and hierarchical clustering. Significant features were then identified using MS/MS. The workflow revealed 24 significant features after 1-phenyl-2-(1-pyrrolidinyl)-1-butanone and 39 after 1-phenyl-2-(1-pyrrolidinyl)-1-heptanone incubation, consisting of adducts, artifacts, isomers, and metabolites. The applied untargeted metabolomics strategy was able to find almost all of the metabolites that were previously described for 1-phenyl-2-(1-pyrrolidinyl)-1-butanone in literature as well as three additional metabolites. Concerning 1-phenyl-2-(1-pyrrolidinyl)-1-heptanone biotransformation in pHLM, merely four metabolites described in primary human hepatocytes and human urine were not found. This study revealed that untargeted metabolomics workflows are well suited to support biotransformation studies at least of the investigated compounds in pHLM.

## Introduction

Knowledge about the metabolism of abused drugs is often essential for a reliable confirmation of an intake by patients in clinical and forensic toxicology^1^. This can especially be the case if metabolites are the only targets for their detection in urine^2^. According to the World Drug Report 2018^3,4^, 803 new psychoactive substances (NPS) were reported between 2009 and 2017. These substances are often legally available while the health risk they entail remains unknown. This implies that continuous metabolism studies are necessary. Due to the lack of authentic human urine, such studies are often performed in advance using different *in vitro* and *in vivo* models such as pooled human liver microsomes (pHLM), pooled human liver S9 fraction (pS9), or rat urine^2,5^. pHLM is the most frequently used *in vitro* model, since it is easily accessible and the majority of drugs undergoes merely cytochrome P450 (CYP) mediated oxidation as well as uridine 5′-diphospho-glucuronosyltransferase mediated glucuronidation^6^. Both enzyme classes are located at the smooth endoplasmic reticulum that is part of the microsomal fraction^5^. The identification of metabolites is then conducted by comparing the fragmentation pattern of the parent compound with that of putative metabolites after mass spectrometry (MS) coupled to liquid (LC)- or gas chromatography (GC)^2,7–9^. Metabolism leads to changes in the chemical structure that can be identified by specific shifts in the fragmentation pattern^1^. This approach of discovering and identifying metabolites relies on the individual skills of the scientist that classifies a compound as a metabolite, based on the experience and knowledge of drug metabolism and interpretation of mass spectra.

The dependency on the experience of individuals is a drawback that might at least be avoided concerning the relevance of an analyte by application of metabolomics techniques, amongst others. Metabolomics in general means the analysis of the whole metabolome (e.g. in urine) and thus aims to detected as many metabolites as possible^10^. In case of human urine this includes metabolites that arise from food, physiological processes, or the gut microbiome^10^ and very likely also may include NPS metabolites. Metabolomics can be divided into two groups. The first one, targeted metabolomics, aims to analyze certain metabolites found relevant and often also quantify their changes. This implies that the metabolites of interest are already known and their relevance has been confirmed. For the investigation of the metabolism of abused drugs (e.g. NPS), such an approach is usually not appropriate since the metabolic pathways still need to be revealed. The identification of relevant metabolites is carried out by untargeted metabolomics (UM). This kind of study provides an impartial approach on every peak that was recorded during the mass spectral analysis. It uses statistical evaluation of the peak abundances to highlight significant changes between two or more investigated groups^11^. After the detection by an algorithm, chromatographic peaks are usually called features that are statistically assessed.

**Figure 1:**
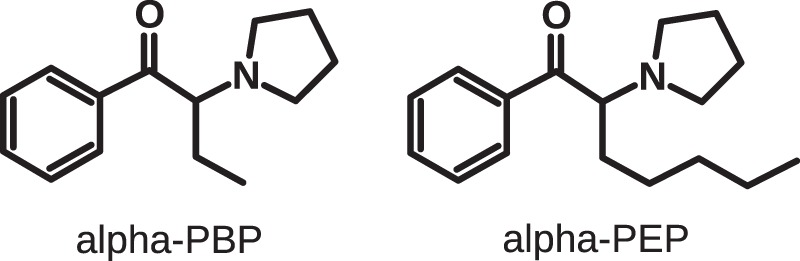
Chemical structures of the investigated compounds.

This study aimed to evaluate untargeted metabolomics strategies for the identification of metabolites of NPS. The evaluation was conducted by investigating the metabolism of the two synthetic cathinones alpha-PBP (1-phenyl-2-(1-pyrrolidinyl)-1-butanone) and alpha-PEP (1-phenyl-2-(1-pyrrolidinyl)-1-heptanone, see Fig. 1) in pHLM incubations and compare the findings to previous metabolism studies^12–14^. Those previous studies revealed that both substances form several CYP-mediated metabolites. Furthermore, glucuronides were found in human urine.

## Experimental

### Chemicals and reagents

alpha-PBP and alpha-PEP were provided by the Bavarian State Criminal Police Office (Munich, Germany). NADP-Na_2_, acetonitrile (LC-MS grade), and methanol (LC-MS grade) were obtained from VWR (Darmstadt, Germany), MgCl_2_, K_2_HPO_4_, K_3_PO_4_, superoxide dismutase, isocitrate dehydrogenase, isocitrate, ammonium formate, ammonium acetate, and formic acid from Sigma (Taufkirchen, Germany). Water was purified with a Millipore filtration unit (18.2 Ω × cm water resistance). pHLM (pool of 25 donors, 20 mg microsomal protein/mL) were obtained from Corning (Amsterdam, The Netherlands). After delivery, the pHLM were thawed at 37 °C, aliquoted, snap-frozen in liquid nitrogen, and stored at −80 °C until use.

### Microsomal incubations

Incubations using pHLM were performed according to Welter *et al*.^15^ alpha-PBP and alpha-PEP were freshly dissolved in 100 mM phosphate buffer and subsequently diluted to obtain the required concentrations in prior to the experiment. For UM, incubations were conducted at 37 °C using 0 (group Blank), 12.5 (group Low), or 25 µM (group High) substrate and 50 mg protein/mL pHLM. The final incubation volume was 50 µL and consisted of 90 mM phosphate buffer, 5 mM isocitrate, 5 mM Mg^2+^, 1.2 mM NADP^+^, 200 U/mL superoxide dismutase, and 0.5 U/mL isocitrate dehydrogenase. The mixture was preincubated for 10 min, reaction was started by the addition of substrate. After 60 min the reaction was stopped by adding 50 µL of ice-cold acetonitrile and the mixture was subsequently centrifuged for 2 min at 14,000 × g. These incubations were performed in five replicates for every group. 10 µL of each incubation were transferred into one MS vial. This pooled sample mixture (group QC) was used for optimization of peak picking and batch correction as described below. 70 µL of the remaining supernatant were transferred into separate MS vials.

For the identification of significant features, incubations were repeated in duplicates using 0 (substrate blank), 12.5, and, 25 µM of substrate. Additionally, incubations were performed not containing pHLM but corresponding amounts of phosphate buffer and 25 µM substrate (enzyme blank). Each of these incubations was conducted as described above.

### LC-HRMS/MS apparatus

The analysis was performed using a Thermo Fisher Scientific (TF, Dreieich, Germany) Dionex UltiMate 3000 RS pump consisting of a degasser, a quaternary pump, and an UltiMate Autosampler, coupled to a TF Q-Exactive Plus system equipped with a heated electrospray ionization HESI-II source. Mass calibration was done prior to analysis according to the manufacturer’s recommendations using external mass calibration. Additionally before each experiment the apparatus’ spray shield and capillary was cleaned. The performance of the column and the mass spectrometer was tested using a test mixture as described by Maurer *et al*.^1,16^ in prior to every experiment. The conditions were set according to published procedures^17,18^. Gradient reversed phase elution was performed on a TF Accucore PhenylHexyl column (100 mm × 2.1 mm, 2.6 µm) or a normal phase Macherey-Nagel (Düren, Germany) HILIC Nucleodur column (125 × 3 mm, 3 μm). The mobile phases for gradient elution using the PhenylHexyl column consisted of 2 mM aqueous ammonium formate containing acetonitrile (1%, v/v) and formic acid (0.1%, v/v, pH 3, eluent A), as well as 2 mM ammonium formate solution with acetonitrile:methanol (1:1, v/v) containing water (1%, v/v) and formic acid (0.1%, v/v, eluent B). The flow rate was set from 1–10 min to 500 µL/min and from 10–13.5 min to 800 µL/min using the following gradient: 0–1.0 min 99% A, 1–10 min to 1% A, 10–11.5 min hold 1% A, 11.5–13.5 min hold 99% A. The gradient elution using the HILIC column was performed using aqueous ammonium acetate (200 mM, eluent C) and acetonitrile containing formic acid (0.1%, v/v, eluent D). The flow rate was set to 500 µL/min using the following gradient: 0–1 min 2% C, 1–5 min 20% C, 5–8.5 min 60% C, 8.5–10 min hold 60% C. 10–12 min hold 2% C. For preparation and cleaning of the injection system isopropanol:water (90:10, v/v) was used. The following settings were used: wash volume, 100 µL; wash speed, 4000 nL/s; loop wash factor, 2. Every analysis was performed at 40 °C column temperature, maintained by a Dionex UltiMate 3000 RS analytical column heater. The injection volume for every analysis was 1 µL. The HESI-II source conditions for every experiment were as follows: ionization mode, positive or negative; sheath gas, 60 AU; auxiliary gas, 10 AU; sweep gas, 3 AU; spray voltage, 3.50 kV in positive mode and −4.0 kV in negative mode; heater temperature, 320 °C; ion transfer capillary temperature, 320 °C; and S-lens RF level, 50.0. Mass spectrometry for UM was performed according to a previously optimized workflow using full scan (FS) only^19^. The settings for FS data acquisition were as follows: resolution, 140,000 fwhm; microscans, 1; automatic gain control (AGC) target, 5 × 10^5^; maximum injection time, 200 ms; scan range, *m/z* 50–750; polarity, negative or positive; spectrum data type, centroid. Significant features were identified using parallel reaction monitoring (PRM). Settings for PRM data acquisition were as follows: resolution, 70,000 fwhm; microscans, 1; AGC target, 5 $\times$ 10^5^; maximum injection time, 200 ms; isolation window, 0.4 m/z; normalized collision energy (NCE), 35 eV; spectrum data type, centroid. The inclusion list contained the monoisotopic masses of all significant features and a time window of their retention time $\pm$30 s. TF Xcalibur software version 3.0.63 was used for data handling. The analysis was performed using a randomized sequence order with five injections of pure methanol (PhenylHexyl column) or eluent D (HILIC column) samples at the beginning of the sequence for apparatus equilibration, followed by five injections of the pooled QC sample. Additionally, one QC injection was performed every five samples to monitor batch effects as described by Wehrens *et al*.^20^.

### Data processing

Thermo Fisher LC-HRMS/MS RAW files were converted into mzXML files using Proteo Wizard^21^. Peak picking was performed using XCMS in an R environment^22,23^, annotation of isotopes, adducts, and artifacts was performed using the R package CAMERA^24^. Optimization of XCMS parameters was in accordance to a previously optimized strategy^19^. Peak picking and alignment parameters are summarized in Table S1. After peak picking, fold changes of features between the groups Blank and Low, Blank and High, as well as Low and High were calculated. A corresponding p-value was calculated using Welsh’s two sample t-test. The data set was filtered keeping merely those features with a fold change <−1.5 or >1.5 and a corresponding p-value of <0.001 in one of the group comparisons. Additionally, every feature with a retention time lower than 60 s or higher than 600 s was removed, since they were acquired during column acquisition or washing phase of the chromatography. According to Wehrens *et al*.^20^ feature abundances with a value of zero were replaced by the lowest measured abundance as a surrogate LOD and subsequently log10 transformed. A batch correction was performed for those features that were detected in every QC sample. The corresponding feature abundance was corrected using a linear model to extrapolate its abundance drift between QC samples^20^. Patterns in the data set were subsequently investigated using principal component analysis (PCA), t-distributed stochastic neighborhood embedding (t-SNE)^25,26^ and hierarchical clustering. Names of the features were adopted from XCMS using “M” followed by the rounded mass and “T” followed by the retention time in seconds (e.g. “M218T216” as given in Table 1 for protonated alpha-PBP at *m/z* 218.1540 and a retention time of 216 s using a PhenylHexyl column).

**Table 1:** Significant features of alpha-PBP after untargeted analysis using a reversed phase (PhenylHexyl) column.

Polarity	Feature	measured mass, *m/z*	retention time, s	Identity
pos	M216T214	216.1383	214	alpha-PBP artifact (dehydro-)
pos	M217T214	217.1415	214	alpha-PBP artifact (dehydro-) ^13^C isotope
pos	M218T216	218.1540	216	alpha-PBP
pos	M219T216	219.1572	216	alpha-PBP ^13^C-isotope
pos	M220T226	220.1695	226	**alpha-PBP-M (dihydro-)**
pos	M221T226	221.1728	226	alpha-PBP-M (dihydro-) ^13^C-isotope
pos	M232T343	232.1331	343	**alpha-PBP-M (oxo-)**
pos	M236T214	236.1643	214	**alpha-PBP-M (dihydro-HO-)**
pos	M237T214	237.1676	214	alpha-PBP-M (dihydro-HO-) ^13^C-isotope
pos	M250T215	250.1435	215	**alpha-PBP-M (di-HO-)**

Features are ordered by *m/z* and retention time. Isotopes were annotated by the R package CAMERA and not further identified. Metabolites are indicated by bold font. pos = positive.

## Results and Discussion

### Untargeted metabolomics

**Figure 2:**
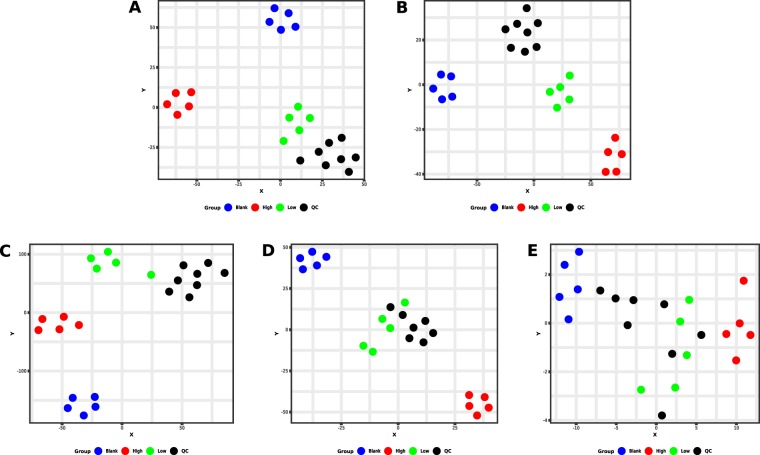
Results of t-SNE. **A** = alpha-PBP, PhenylHexyl column, positive mode; **B** = alpha-PBP, HILIC column, positive mode; **C** = alpha-PEP, PhenylHexyl column, positive mode; **D** = alpha-PEP, HILIC column, positive mode; **E** = alpha-PEP, HILIC column, negative mode.

**Figure 3:**
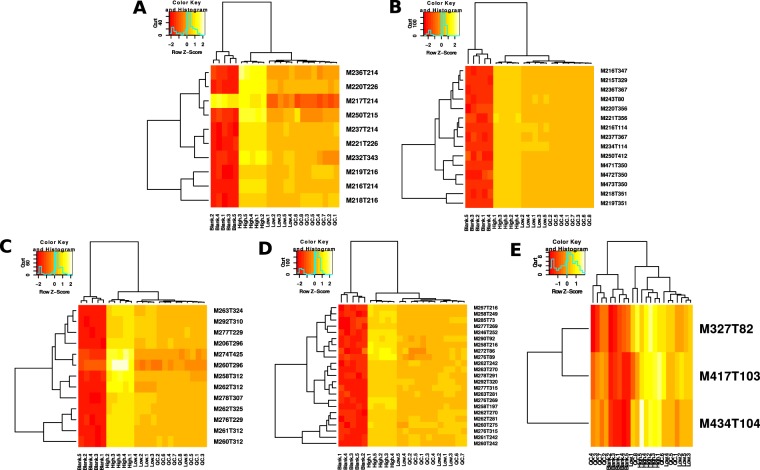
Heat maps of hierarchical clustering. **A** = alpha-PBP, PhenylHexyl column, positive mode; **B** = alpha-PBP, HILIC column, positive mode; **C** = alpha-PEP, PhenylHexyl column, positive mode; **D** = alpha-PEP, HILIC column, positive mode; **E** = alpha-PEP, HILIC column, negative mode.

Volcano plots of detected features are shown in Figures S1–S8, Scores and Loadings of the PCA in Figures S9 and S10. Results of t-SNE and heat maps of hierarchical clustering are presented in Figs 2 and 3. The first of five QC samples that were analyzed before samples from the different incubation groups during the analysis of alpha-PEP using HILIC and positive ionization mode had to be excluded due to a high sensitivity loss of the MS during the analysis. Almost none of the analyses using negative mode revealed significant features. An exception was the analysis of alpha-PEP in HILIC/negative mode (Figure S8). PCA revealed that more than 95% of the variance was stored in the first principal component leading to the conclusion that the data is characterized by a high amount of collinearity (Fig. S9A–D). This is not a surprise regarding the high amount of parent compound in relation to other substances within the incubation mixture. Samples of the group Blank also persistently displayed a high variability within their group throughout most of the analyses indicating a high variance in the measured peak abundances. Such high variance is also explainable by the study design since the variance in the data set primarily originated from the addition of parent compound and the subsequent formation of metabolites. If a peak was detected and integrated by XCMS it also reintegrated the same region in other samples where they were originally not detected. Regarding that samples of the group Blank did not contain any parent compound or metabolite, XCMS merely integrated noise that resulted in peak areas with a high variance between different samples which is visible in these score plots. An exception was the analysis of alpha-PEP using HILIC in negative mode (Fig. S9E). Since neither the parent compounds nor their metabolites did contain any structural elements such as halogen atoms or carboxylic acids that can be ionized in negative mode under the given conditions, the above-mentioned restrictions did not apply here. The corresponding loadings (Figure S10 E) showed that the three features that were found significant in this analysis contributed evenly to the group separation.

These findings implied that even though PCA is a widely used technique for untargeted metabolomics, it was not suitable for those experiments where the substrate and its metabolites were detectable. Patterns in the data were therefore investigated using t-SNE, a dimension reduction algorithm that visualized similarities in the data set^26^. The t-SNE plots resulted in a more appropriate clustering of the groups (Fig. 1) with evenly distributed distances. QC samples appear very close or within the cluster of samples from the group Low. This is not surprising since the pooled sample QC consists of a mixture of every incubation sample and thus contains parent compound and its metabolites in an approximate concentration of the samples from group Low. Differences between both group Low and QC might appear due to inaccuracy when preparing the pooled sample QC or a nonlinear metabolite formation in the given concentrations during incubation.

Clustering patterns of t-SNE corresponded to those obtained by hierarchical clustering (Fig. 2). Every analysis in positive mode showed a clear discrimination between samples from the group Blank and the remaining ones. The only exception again was the analysis of alpha-PEP using HILIC (Fig. 2E), were QC samples appeared in the same class as samples from the group Blank. The corresponding z-scores indicated a correlation of the features’ peak abundances with the clustering. It is noteworthy that in the analysis of alpha-PBP in positive mode using the PhenylHexyl column (Fig. 2A), the feature M217T214 showed a positive z-score in the groups Blank and High, but a negative z-score in the groups Low and QC what implied a corresponding decrease of the peak abundances. However, this feature was annotated by CAMERA as a ^13^C isotope (see Table 1) whose peak abundance should increase with increasing concentrations of the parent compound. Inspection of the chromatograms as detected by XCMS revealed a coeluting peak and the beginning of a baseline drift^27^ that artificially increased the peak abundance of those peaks from the group Blank.

### Identification of significant features

The results of the identification of significant features is summarized in Tables 1–4. Isotopes that were annotated by CAMERA were not further analyzed. Every other feature was analyzed as described above using a PRM method and the resulting mass spectra are shown in Figures S9-S13. The Metabolomics Standards Initiative proposed minimum information that need to be supplied when reporting metabolomics experiments^28^. These include a classification of the identification level by four categories. The first category is the most valid identification level using two orthogonal data sets. The last category means an unidentified metabolite. Due to the lack of reference compounds, the proposed structural formulas were deduced by comparing the spectra of the metabolites with those of the parent compounds or reference spectra using the METLIN and Human Metabolome Database (HMDB)^28,29^. This approach is referred to as category two in the above mentioned publication, meaning a putatively annotated compound. It applies to all of the identified compounds except for M417T103 and M434T104 in Table 4 which are yet unknown and therefore category four. The fragmentation of both substances was already described in previous publications^14^ and will not further be discussed here. Concerning the incubations using alpha-PBP, significant features consisted of six isotopes, three artifacts, five metabolites, one adduct, and one impurity. alpha-PBP itself formed one in-source artifact (M216T214 in Table 1 and M216T347 in Table 2). It was the result of an oxidation of the pyrrolidine ring. The formation of the artifact within the ion source was assumed since its retention time was similar to that of the parent compound. The oxidation of the pyrrolidine ring was additionally found at a different retention time using HILIC (M216T114 in Table 2) implying that this compound was not formed within the ion source. Since it was also contained in the enzyme blank but not in the substrate blank sample it is very likely that this compound is an impurity of the powder that was seized by the Bavarian State Criminal Police Office. In addition to the in-source artifact there were two further compounds that are presumably artifacts of different origin (M215T328 and M243T80 in Table 2). The first one contains an imide group instead of a cathinone oxo group and an oxidized pyrrolidine ring. The introduction of an imide could theoretically be conducted by the inversion of a deamination reaction but such a metabolism step has never been reported before and was therefore classified as an artifact of the dehydro impurity. The second artifact contains a cyano group which cannot be introduced by human enzymes. The formation of this artifact might have been occurred within the ion source while using acetonitrile as an eluent or after terminating the incubation with acetonitrile. One dihydro metabolite (M220T355 in Table 2) was found, that was not sufficiently separated from the alpha-PBP isotope containing two ^13^C atoms during the analysis using HILIC. The result of the PRM analysis was therefore a mixed MS^2^ spectrum containing fragments from both parent ions (see Fig. S10). The separation of both compounds was possible after using an alternative chromatography with a flatter increase of eluent D. The employed gradient as well as the obtained chromatogram and MS^2^ spectra are displayed in Figure S14.

**Table 2:** Significant features of alpha-PBP after untargeted analysis using a normal phase (HILIC) column.

Polarity	Feature	measured mass, *m/z*	retention time, s	Identity
pos	M215T328	215.1542	328	alpha-PBP impurity (dehydro-) artifact (imido-)
pos	M216T114	216.1383	114	alpha-PBP impurity (dehydro-)
pos	M216T347	216.1383	347	alpha-PBP artifact (dehydro-)
pos	M218T351	218.1541	351	alpha-PBP
pos	M219T350	219.1572	350	alpha-PBP ^13^C-isotope
pos	M220T355	220.1694	355	**alpha-PBP-M (dihydro-)**
pos	M221T355	221.1726	355	alpha-PBP-M (dihydro-) ^13^C-isotope
pos	M234T114	234.1488	114	**alpha-PBP-M (dihydro-oxo-)**
pos	M236T367	236.1643	367	**alpha-PBP-M (dihydro-HO-)**
pos	M237T367	237.1677	367	alpha-PBP-M (dihydro-HO-) ^13^C-isotope
pos	M243T80	243.1492	80	alpha-PBP impurity (dehydro-) artifact (cyano-)
pos	M250T412	250.1436	412	**alpha-PBP-M (di-HO-)**
pos	M471T350	471.2771	350	alpha-PBP adduct [2 M + 2 H + Cl]^+^
pos	M472T350	472.2805	350	alpha-PBP adduct [2 M+2 H+Cl]^+ 13^C-isotope
pos	M473T350	473.2742	350	alpha-PBP adduct [2 M+2 H+Cl]^+ 13^C_2_-isotope

Features are ordered by *m/z* and retention time. Isotopes were annotated by the R package CAMERA and not further identified. Metabolites are indicated by bold font. pos = positive.

**Table 3:** Significant features of alpha-PEP after untargeted analysis using a reversed phase (PhenylHexyl) column.

Polarity	Feature	measured mass, *m/z*	retention time, s	Identity
pos	M206T296	206.1538	296	**alpha-PEP-M (N**,**N-dealkyl-)**
pos	M258T312	258.1852	312	alpha-PEP artifact (dehydro-)
pos	M260T296	260.2008	296	alpha-PEP conformer 2
pos	M260T312	260.2009	312	alpha-PEP conformer 1
pos	M261T312	261.2042	312	alpha-PEP ^13^C-isotope
pos	M262T312	262.2073	312	alpha-PEP ^13^C_2_-isotope
pos	M262T325	262.2165	325	**alpha-PEP-M (dihydro-)**
pos	M263T324	263.2197	324	alpha-PEP-M (dihydro-) ^13^C-isotope
pos	M274T425	274.1799	425	**alpha-PEP-M (oxo-)**
pos	M276T229	276.1958	229	**alpha-PEP-M (HO-) isomer 2**
pos	M277T229	277.1990	229	alpha-PEP-M (HO-) isomer 2 ^13^C-isotope
pos	M278T307	278.2113	307	**alpha-PEP-M (dihydro-HO-)**
pos	M292T310	292.1904	310	**alpha-PEP-M (di-HO-)**

Features are ordered by *m/z* and retention time. Isotopes were annotated by the R package CAMERA and not further identified. Metabolites are indicated by bold font. pos = positive.

**Table 4:** Significant features of alpha-PEP after untargeted analysis using a normal phase (HILIC) column.

Polarity	Feature	measured mass, *m/z*	retention time, s	Identity
pos	M246T254	246.1849	254	alpha-PHP impurity
pos	M257T216	257.2008	216	alpha-PEP impurity (dehydro-) artifact (imido-)
pos	M258T198	258.1849	198	alpha-PEP impurity (dehydro-)
pos	M258T216	258.2042	216	alpha-PEP impurity (dehydro) artifact (imido-) C^13^-isotope
pos	M258T249	258.1849	249	alpha-PEP artifact (dehydro-)
pos	M260T243	260.2007	243	alpha-PEP conformer 1
pos	M260T276	260.2005	276	alpha-PEP conformer 2
pos	M261T243	261.2040	243	alpha-PEP ^13^C-isotope
pos	M262T243	262.2071	243	alpha-PEP ^13^C_2_ isotope
pos	M262T271	262.2162	271	**alpha-PEP-M (dihydro-) diastereomer 1**
pos	M262T282	262.2162	282	**alpha-PEP-M (dihydro-) diastereomer 2**
pos	M263T270	263.2195	270	alpha-PEP-M (dihydro-) diastereomer 1 ^13^C-isotope
pos	M263T282	263.2195	282	alpha-PEP-M (dihydro-) diastereomer 2 ^13^C-isotope
pos	M272T87	272.1641	87	alpha-PEP-M (oxo-HO-) isomer 2 artifact
pos	M276T90	276.1954	90	**alpha-PEP-M (dihydro-oxo-)**
pos	M276T269	276.1954	269	**alpha-PEP-M (HO-) isomer 1**
pos	M276T316	276.1954	316	**alpha-PEP-M (HO-) isomer 2**
pos	M277T269	277.1987	269	alpha-PEP-M (HO-) isomer 1 ^13^C-isotope
pos	M277T316	277.1987	316	alpha-PEP-M (HO-) isomer 2 ^13^C-isotope
pos	M278T291	278.2110	291	**alpha-PEP-M (dihydro-HO-)**
pos	M285T74	285.1957	74	alpha-PEP impurity (dehydro-) artifact (cyano-)
pos	M290T93	290.1746	93	**alpha-PEP-M (oxo-HO-) isomer 1**
pos	M292T321	292.1901	321	**alpha-PEP-M (di-HO-)**
neg	M327T82	327.2323	82	Docosahexaenoic acid
neg	M417T103	417.1910	103	Unknown
neg	M434T104	434.1811	104	Unknown

Features are ordered by *m/z* and retention time. Isotopes were annotated by the R package CAMERA and not further identified. Metabolites are indicated by bold font. pos = positive, neg = negative.

The significant features identified in the incubations using alpha-PEP consisted of seven isotopes, four artifacts, nine metabolites, two impurities, one endogenous compound, and two yet unidentified compounds. For the parent compound of alpha-PEP two features with different abundances were found showing the same fragmentation but different abundances of the corresponding fragments (M260T312 and M260T296 in Table 3, as well as M260T243 and M260T276 in Table 4). A considerable difference between the MS^2^ spectra is the huge intensity of the fragment at *m/z* 242.1901 in Fig. S11 and *m/z* 242.1903 in Fig. S12. This fragment occurs after the initial elimination of the cathinone oxo group and leads to the conclusion that these conformers arise from a different conformation of the pyrrolidine ring in relation to the oxo group. The analysis using HILIC revealed two dihydro metabolites of alpha-PEP (M262T271 and M262T282 in Table 4). Since the reduction of the cathinone oxo group introduces an additional stereo center into the molecule, these two features are most likely different diastereomers of the same compound. Due to an insufficient separation, the analysis using a PhenylHexyl column merely revealed one feature identified as a dihydro metabolite (M262T325 in Table 3). One of the artifacts that were found in the analysis of alpha-PEP was also an in-source artifact of the parent compound and followed the same formation principle as that of alpha-PBP (M258T312 in Table 3, M258T249 in Table 4). An impurity with an oxidized pyrrolidine ring was also found (M246T254 in Table 4), forming the same artifacts as already described for the analysis of alpha-PBP (M257T216 and M285T74 in Table 4). Another feature was identified as compound with a shorter alkyl chain (M246T254in Table 4). Alkyl chain shortening may be a result of human fatty acid like metabolism. The the β-oxidation usually results in carboxylic acids that are further broken down by subsequent oxidation steps. However, the identified compound did not contain such structural elements leading to the conclusion that it might also be an impurity.

The analysis of alpha-PEP using HILIC and negative ionization mode revealed three significant features. The fragmentation of these features could not be explained by using the parent compound fragmentation leading to the assumption that these features might be endogenous compounds. For further identification, the METLIN and the HMDB were searched using the MS/MS search method of both databases and the respective MS^2^ spectra. The precursor *m/z* deviation was set to a maximum of 5 ppm, the *m/z* deviation of the corresponding fragments was set to 0.01 Da for METLIN and 10 ppm for HMDB. One feature (M327T82 in Table 4) was identified as Docosahexaenoic acid (Metlin ID: 3457, HMDB0002183) with a METLIN score of 58 and a HMDB fit of 85%. Both databases matched the compound to experimental MS/MS data. Docosahexaenoic acid is an important structural component of the human brain, cerebral cortex, and the discs of the rod photoreceptor cells. It can be provided via animal fats or converted from alpha-linolenic acid and linoleic acid in the human liver^30^. Since docosahexaenoic acid was the only lipid that was found in this study the cause of its enrichment in incubations with alpha-PEP remains speculative. The remaining features (M417T103 and M434T104 in Table 4) are yet unknown since their spectra did not lead to any match in the above-mentioned databases. Their MS^2^ spectra needed to be recorded using an isolation window of *m*/*z* 1 to obtain sufficient scan points. Since they could not be identified, their spectra were additionally recorded using an isolation window of *m/z* 1 to obtain sufficient scan points and collision energies of 10, 20, and 40 eV. Mass spectra were included in Figure S13 without the annotation of a putative composition and a corresponding ppm deviation.

### Metabolism of alpha-PBP and alpha-PEP in pHLM

**Figure 4:**
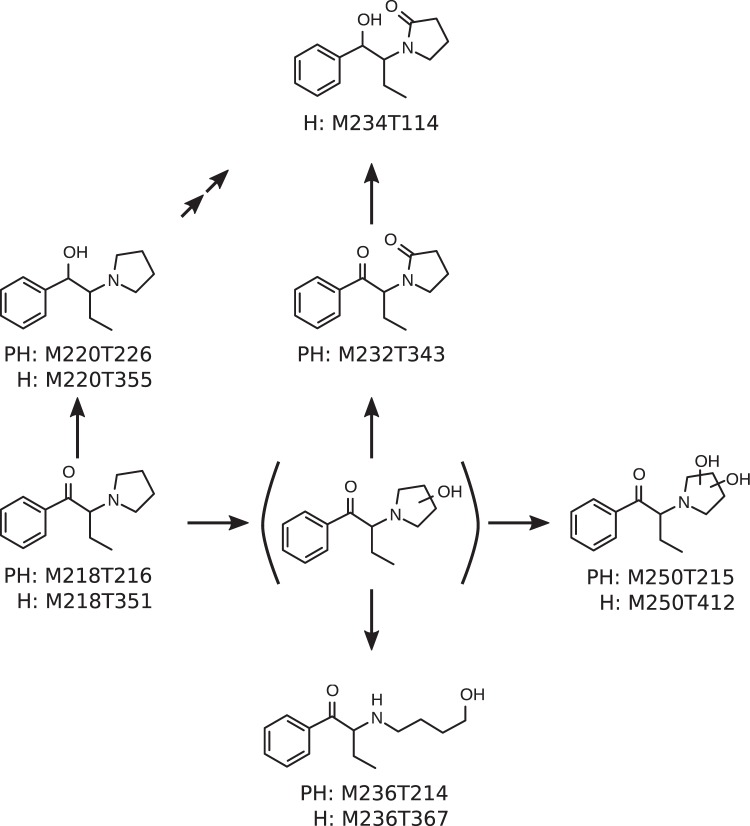
Metabolic pathways of alpha-PBP in pHLM. Undefined position of hydroxylation indicated by unspecific bonds. Two arrows indicate a pathway that contains multiple metabolism steps. Every metabolite is annotated with its feature identifier from untargeted metabolomics analyses. PH = PhenylHexyl column, H = HILIC column.

**Figure 5:**
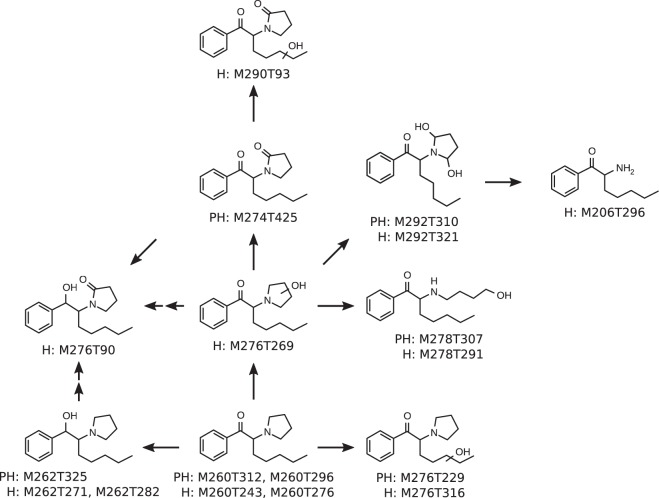
Metabolic pathways of alpha-PEP in pHLM. Undefined position of hydroxylation indicated by unspecific bonds. Two arrows indicate a pathway that contains multiple metabolism steps. Every metabolite is annotated with its feature identifier from untargeted metabolomics analyses. PH = PhenylHexyl column, H = HILIC column.

The metabolic pathways of alpha-PBP and alpha-PEP in pHLM as elucidated by this study are represented by Figs 4 and 5. alpha-PBP was reduced at the cathinone oxo group resulting in a dihydro metabolite. The pyrrolidine ring underwent metabolism resulting in an oxo and a dihydroxy metabolite as well as a ring opened hydroxy metabolite. The oxo group was suggested to be in ortho position due to the retention time of the metabolite. The oxo metabolite was eluting later than the parent compound using a PhenylHexyl column and earlier using HILIC. This allowed the conclusion that this compound should be more lipophilic. Higher lipophilic properties of oxo metabolites can be explained by the existence of a lactam. This finding is in accordance to previous publications investigating the metabolism of alpha-cathinones and one study describing the biotransformation of nitrogen containing xenobiotics to lactams^13,31–34^. The opening of the pyrrolidine ring is very likely the result of a hydroxylation in ortho position of the pyrrolidine ring followed by a retro-hemiaminal reaction. The resulting aldehyde was finally reduced forming the hydroxy group. Additionally, a combination of the dihydro and the oxo metabolite was found. The metabolism of alpha-PBP in pHLM and pS9 has already been investigated by Manier *et al*., as well as in human urine by Matsuta *et al*.^12,14^. Most metabolites found in this study were already described in these publications. Nevertheless, the UM approach was able to additionally identify a di-hydroxy metabolite, a ring opened dihydro hydroxy metabolite, as well as a dihydro oxo metabolite. The dihydro metabolite was not found in previous pHLM and pS9 investigations but in human urine. This might be due to the case, that the dihydro metabolite showed a similar retention time as the parent compound when using a PhenylHexyl column and is thus hard to distinguish from the parent compound isotope containing two ^13^C atoms. The above described mass spectrometer settings with a resolution of 140,000 fwhm however allowed to distinguishing both peaks. Matsuta *et al*. were able to separate the dihydro metabolite from the parent compound since they used gas chromatography^12^. The hydroxy metabolite described by Manier *et al*. in previous studies was not found by the metabolomics approach used in this study. Considering that Matsuta *et al*. did not find a hydroxy metabolite in human urine^12^ leads to the assumption that this metabolite is not a primary target and is formed only in very small amounts *in vitro*, which might explain the non-detection via automated peak picking.

alpha-PEP also underwent reduction of the cathinone oxo group resulting in a dihydro metabolite. The pyrrolidine ring was metabolized resulting in an oxo, two hydroxy, and one dihydroxy metabolite. The position of the oxo group was again determined in ortho to the pyrrolidine nitrogen due to the above-mentioned characteristics. The position of the hydroxy groups in the dihydroxy metabolite was determined as vicinal to the nitrogen in the pyrrolidine ring since it is likely that this metabolite leads to the *N*,*N*-dealkyl metabolite that was also detected. In addition to these pathways, alpha-PEP was hydroxylated at the alkyl chain leading to a mono hydroxy metabolite as well as a hydroxylated oxo metabolite. The formation of a ring opened hydroxy metabolite was found as well and likely resulted from the metabolism steps already described for alpha-PBP.

Previous investigations on the metabolism of alpha-PEP were performed by Manier *et al*. using pHLM and pS9 as well as Swortwood *et al*. using primary human hepatocytes (PHH) and human urine^13^. The metabolomic approach after pHLM incubation used in this study was able to reveal all of the metabolites previously found after incubations using pHLM and additionally most of those found in pS9, PHH, and human urine. Metabolites that were not found by the metabolomic approach were primarily those that resulted from dehydrogenation, as well as the carboxylic acid that likely resulted from hydrolysis of the lactam metabolite. This might have been the result of limitations of the chosen pHLM model in comparison to PHH and human urine.

## Conclusions

*In vitro* metabolism studies using pHLM in combination with untargeted metabolomics resulted in the detection of 24 significant features for alpha-PBP and 39 significant features for alpha-PEP. Most of these features were artifacts, isotopes, conformers, diastereomers, or adducts. Nevertheless, metabolites were identified that corresponded well with those from previous investigations using pHLM, pS9, PHH, and human urine^12–14^. For alpha-PBP three metabolites were found that were not described in any of the previous publications. Investigations of alpha-PEP in pHLM revealed known metabolites but missed those additionally found in PHH and human urine, which is most likely due to enzymatic differences between pHLM and the other models. Besides metabolites of the parent compounds, this study was also able to reveal changes within the incubation mixture after exposure to the drugs. After the incubation of alpha-PEP, docosahexaenoic acid was found enriched, while two yet unidentified compounds showed a significant decrease in their peak abundances. This study demonstrated that the developed untargeted metabolomics approach allows detection of *in vitro* metabolites of NPS after pHLM incubation besides the detection of further compounds by using an investigator independent approach.

## Supplementary information

Electronic Supplementary Material TableS1 and FiguresS1-S16

## Data Availability

Datasets generated during and/or analyzed during the current study are not publicly available but are available from the corresponding author on reasonable request.
