# Supplementary material for: Untargeted metabolomics by high resolution mass spectrometry coupled to normal and reversed phase liquid chromatography as a tool to study the in vitro biotransformation of new psychoactive substances
Source: Sci Rep. 2019 Feb 26;9:2741. doi: 10.1038/s41598-019-39235-w (PMC6391464; doi:10.1038/s41598-019-39235-w)
Supplement: Supplementary file 1 — Electronic Supplementary Material TableS1 and FiguresS1-S16 [file 41598_2019_39235_MOESM1_ESM.pdf]

Electronic Supplementary Material

Untargeted metabolomics by high resolution mass spectrometry coupled to normal and reversed phase liquid chromatography as a tool to study the in vitro biotransformation of new psychoactive substances

Sascha K. Manier, Andreas Keller, Jan Schäper, Markus R. Meyer

Table S1. Peak picking and alignment parameters used for preprocessing. PH = PhenylHexyl, H = HILIC, pos = positive, neg = negative, ppm = allowed ppm deviation of mass traces for peak picking, snthresh = signal to noise threshold, mzdiff = minimum difference in *m*/*z* for two peaks to be considered as separate, prefilter 1 = minimum of scan points, prefilter 2 = minimum abundance, bw = bandwidth for grouping of peaks across separate chromatograms

Compound	Column	Polarity	peakwidth, min	peakwidth, max	ppm	snthresh	mzdiff	prefilter 1	prefilter 2	bw
alpha-PBP	PH	pos	8.9	12	1	90	-0.09	6	4700	0.5
alpha-PBP	PH	neg	6.8	10	1	100	0.074	5	9800	0.5
alpha-PBP	H	pos	10	16	1.4	99	0.078	15	8400	0.5
alpha-PBP	H	neg	9.9	47	1.4	38	0.05	15	9500	0.5
alpha-PEP	PH	pos	6	48	2	77	0.006	29	7000	0.3
alpha-PEP	PH	neg	9	27	1.1	63	0.066	9	9000	0.1
alpha-PEP	H	pos	7.3	31	1.4	43	0.018	17	50000	0.5
alpha-PEP	H	neg	9.9	26	1.6	32	0.04	10	600	0.4

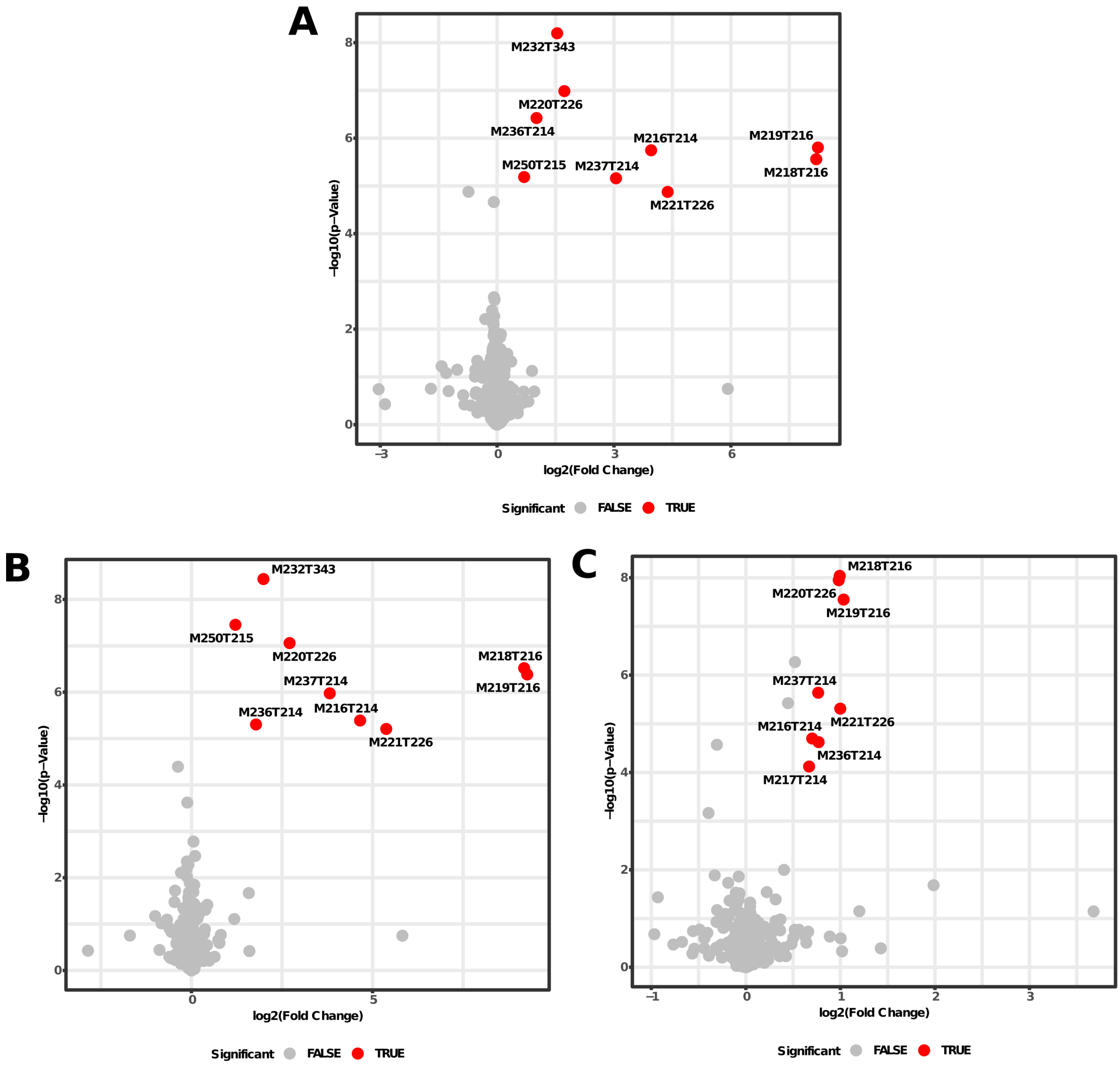

Figure S1. Volcano plots of features detected after analysis of alpha-PBP using a PhenylHexyl column in positive mode. p-value was calculated using Welsh's two sample t-test. Those features with a fold change < 0.5 or > 1.5 and a corresponding p-value < 0.001 were classified as significant. A = Blank vs. Low, B = Blank vs. High, C = Low vs. High

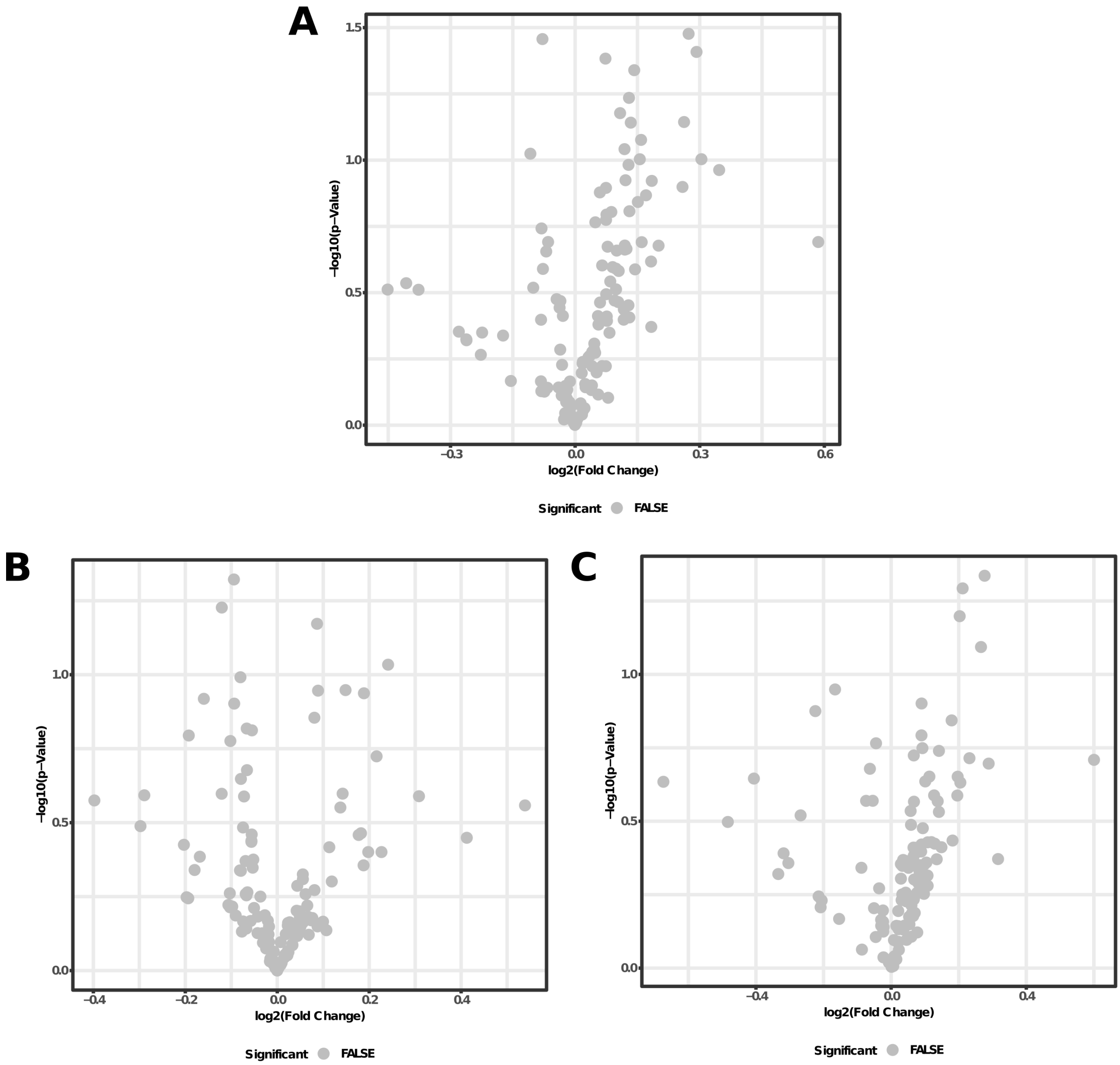

Figure S2. Volcano plots of features detected after analysis of alpha-PBP using a PhenylHexyl column in negative mode. p-value was calculated using Welsh's two sample t-test. Those features with a fold change < 0.5 or > 1.5 and a corresponding p-value < 0.001 were classified as significant. A = Blank vs. Low, B = Blank vs. High, C = Low vs. High

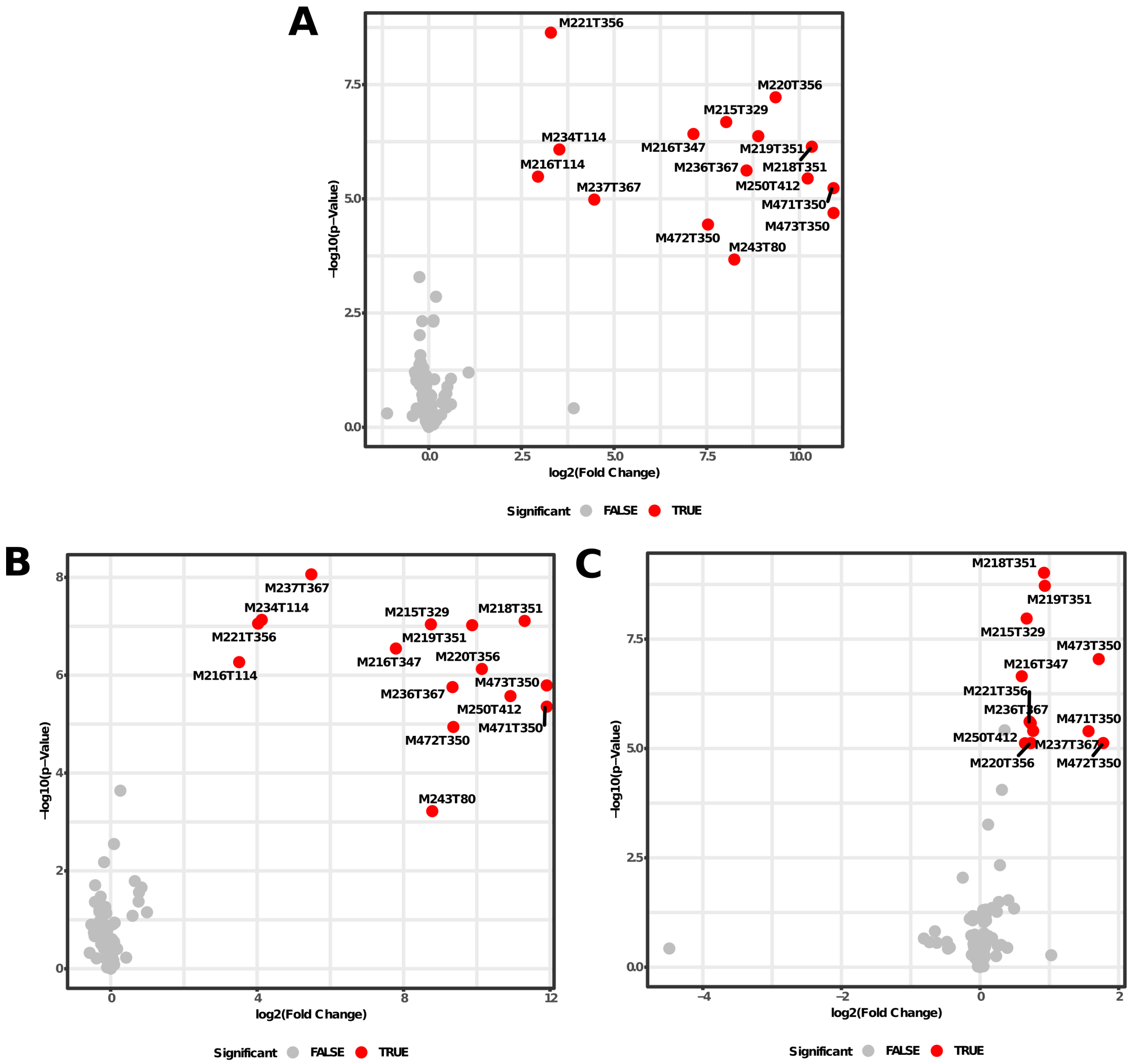

Figure S3. Volcano plots of features detected after analysis of alpha-PBP using a HILIC column in positive mode. p-value was calculated using Welsh's two sample t-test. Those features with a fold change < 0.5 or > 1.5 and a corresponding p-value < 0.001 were classified as significant. A = Blank vs. Low, B = Blank vs. High, C = Low vs. High

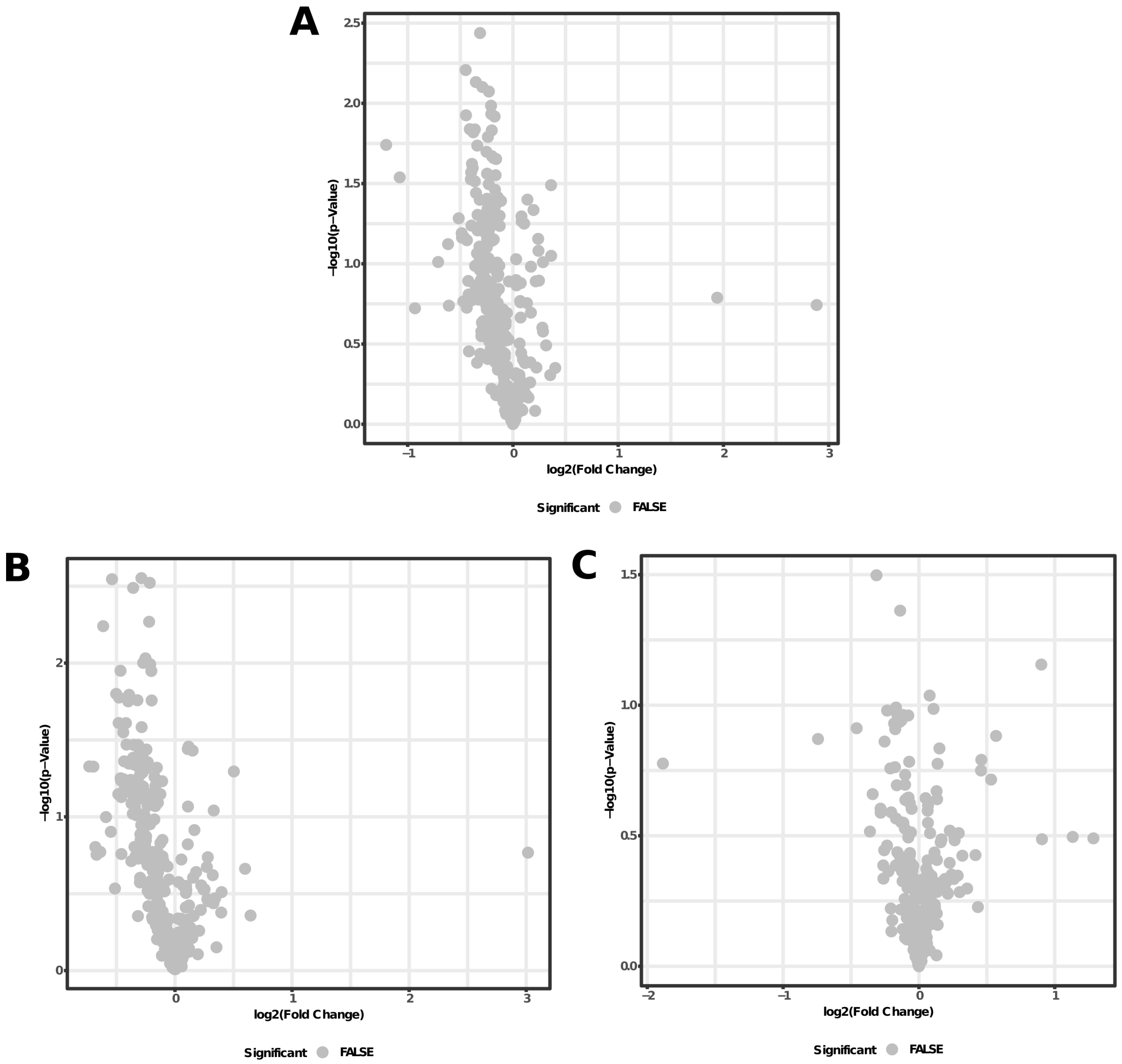

Figure S4. Volcano plots of features detected after analysis of alpha-PBP using a HILIC column in negative mode. p-value was calculated using Welsh’s two sample t-test. Those features with a fold change < 0.5 or > 1.5 and a corresponding p-value < 0.001 were classified as significant. A = Blank vs. Low, B = Blank vs. High, C = Low vs. High

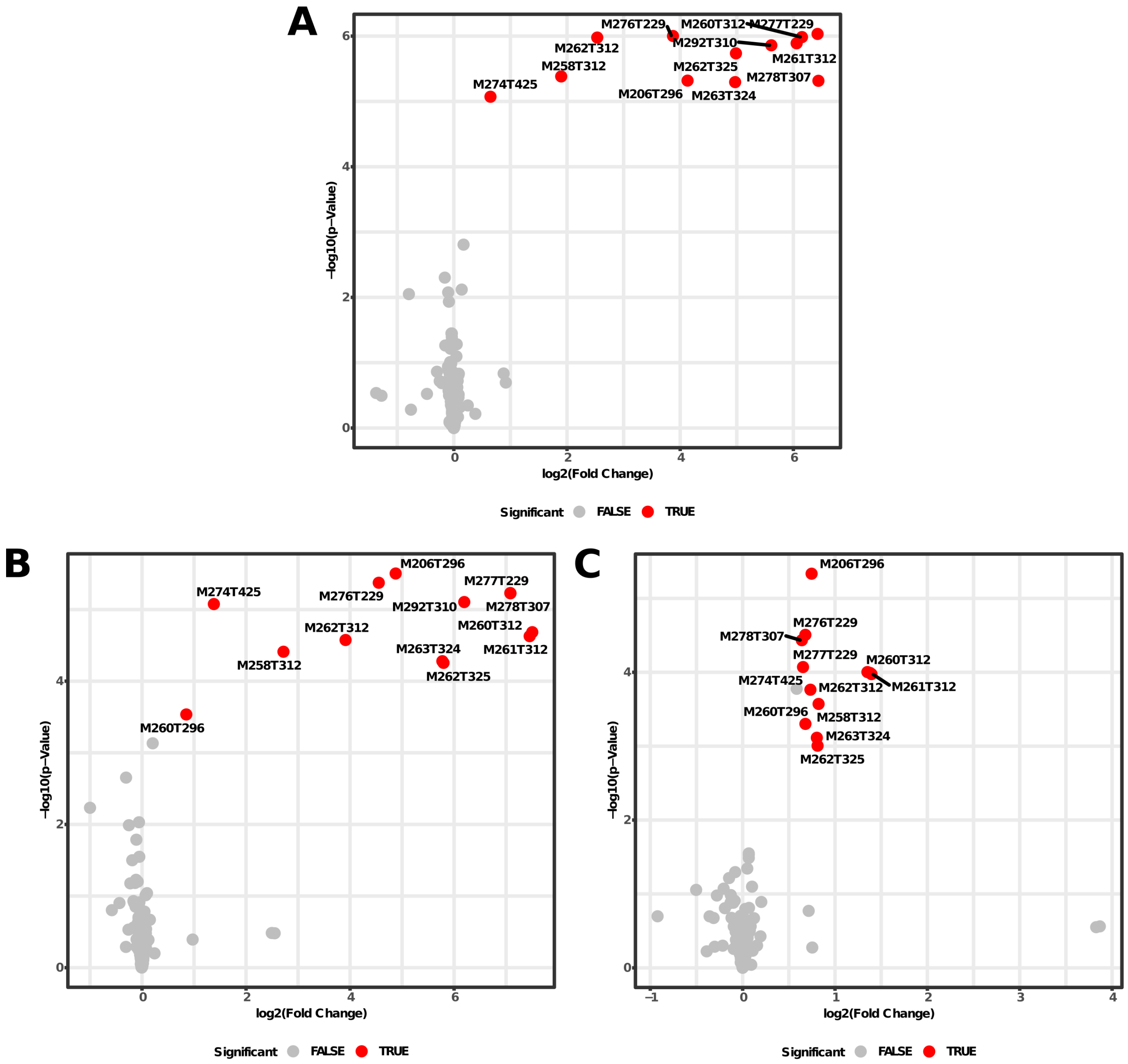

Figure S5. Volcano plots of features detected after analysis of alpha-PEP using a PhenylHexyl column in positive mode. p-value was calculated using Welsh's two sample t-test. Those features with a fold change < 0.5 or > 1.5 and a corresponding p-value < 0.001 were classified as significant. A = Blank vs. Low, B = Blank vs. High, C = Low vs. High

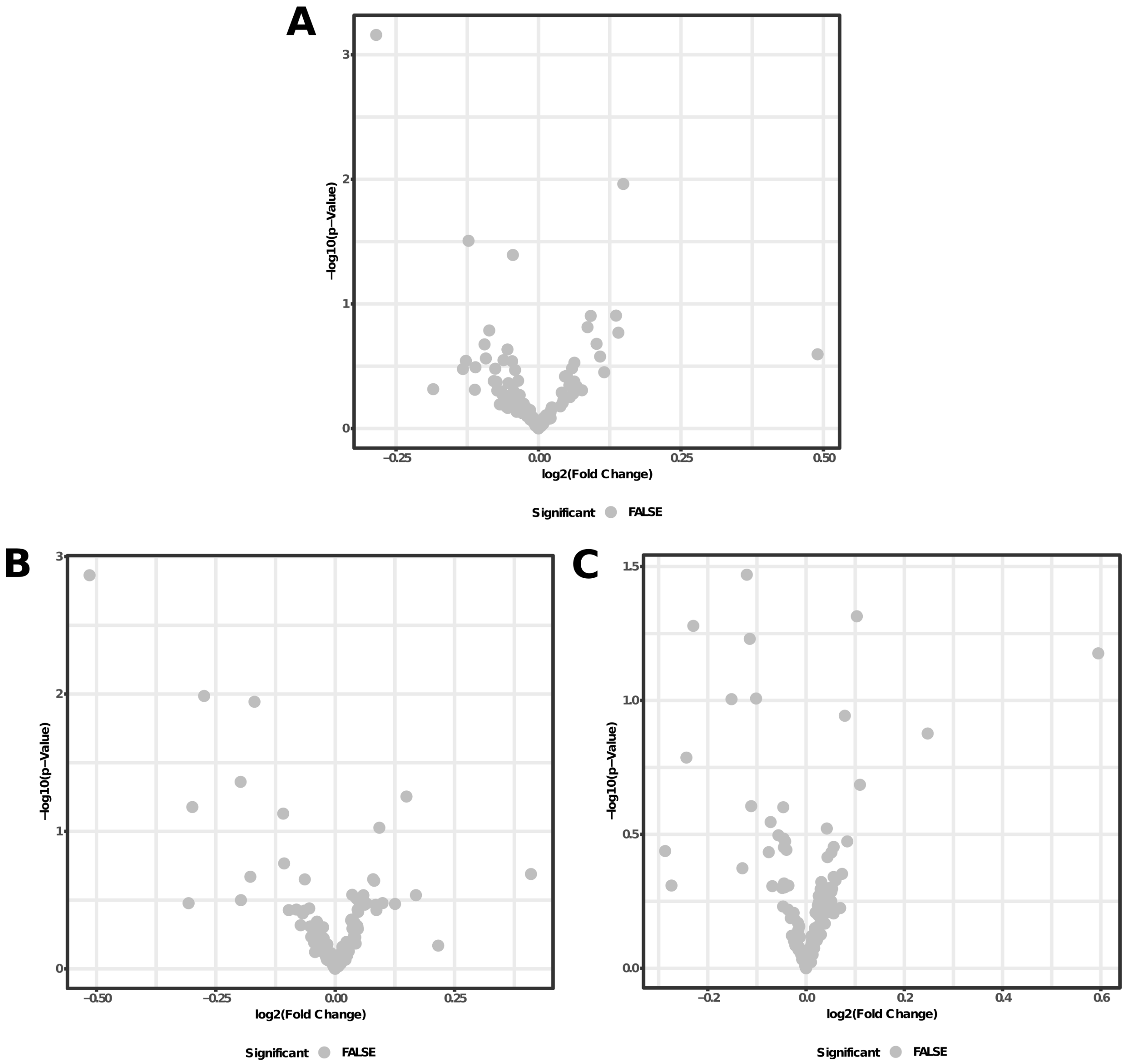

Figure S6. Volcano plots of features detected after analysis of alpha-PEP using a PhenylHexyl column in negative mode. p-value was calculated using Welsh's two sample t-test. Those features with a fold change < 0.5 or > 1.5 and a corresponding p-value < 0.001 were classified as significant. A = Blank vs. Low, B = Blank vs. High, C = Low vs. High

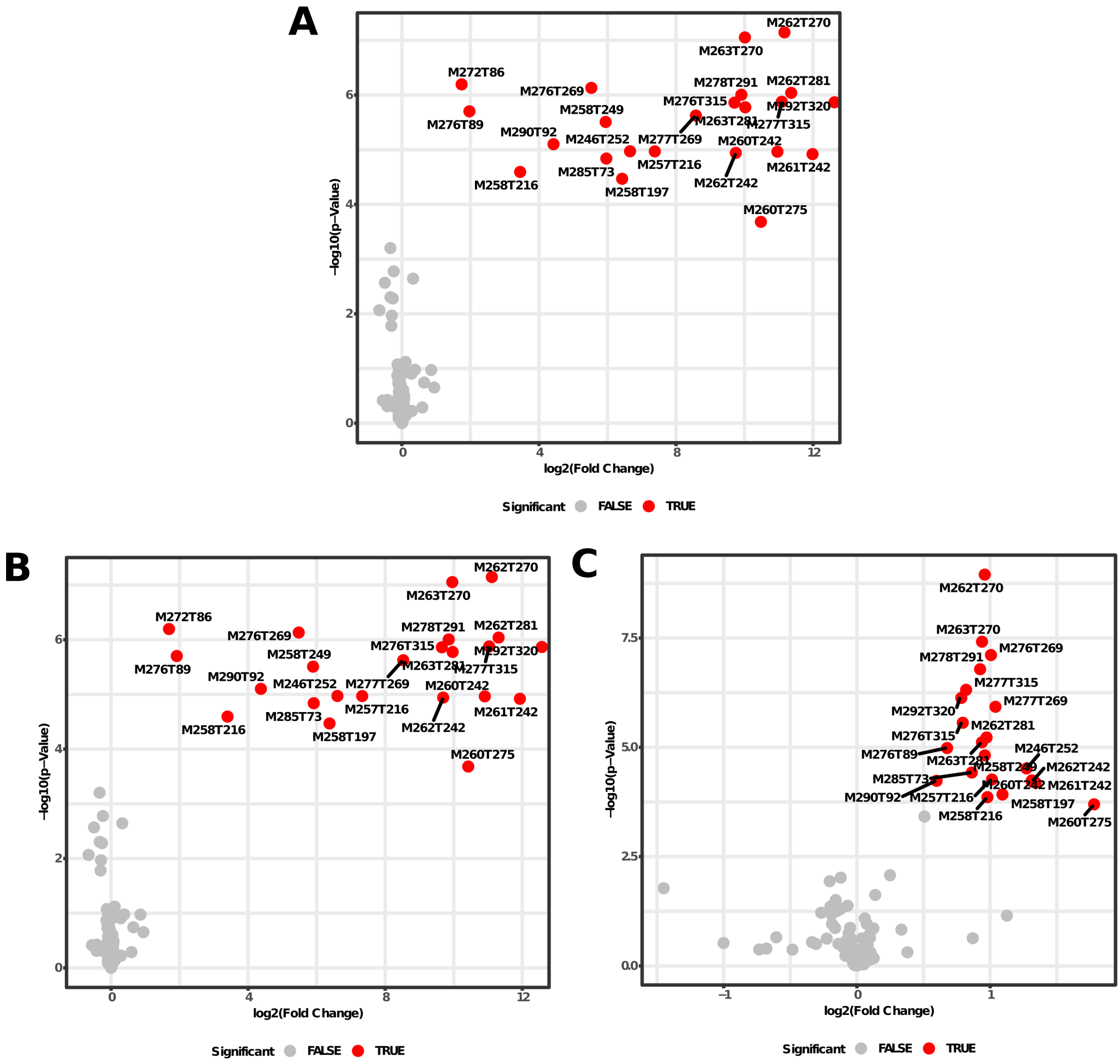

Figure S7. Volcano plots of features detected after analysis of alpha-PEP using a HILIC column in positive mode. p-value was calculated using Welsh's two sample t-test. Those features with a fold change < 0.5 or > 1.5 and a corresponding p-value < 0.001 were classified as significant. A = Blank vs. Low, B = Blank vs. High, C = Low vs. High

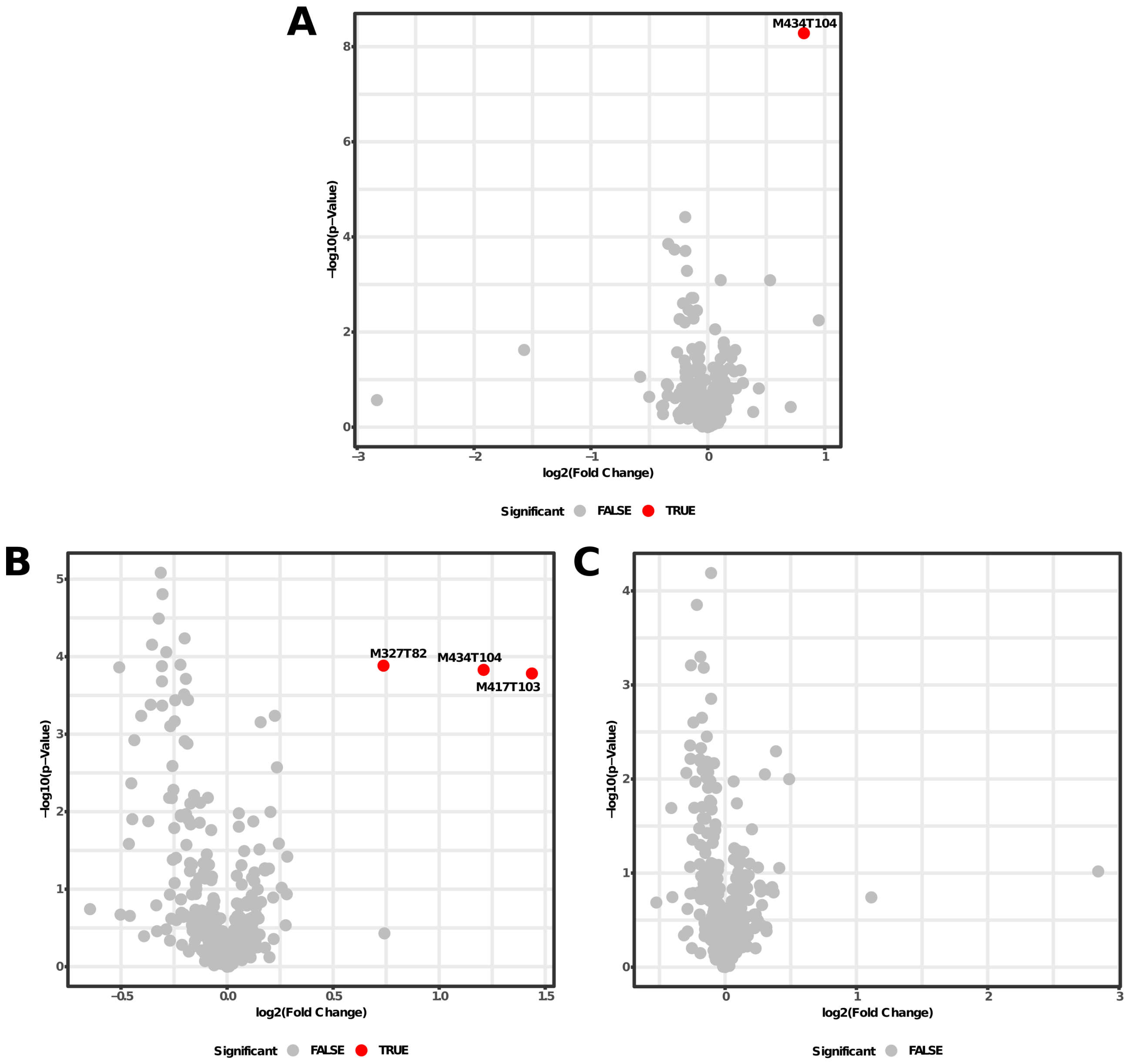

Figure S8. Volcano plots of features detected after analysis of alpha-PEP using a HILIC column in negative mode. p-value was calculated using Welsh's two sample t-test. Those features with a fold change < 0.5 or > 1.5 and a corresponding p-value < 0.001 were classified as significant. A = Blank vs. Low, B = Blank vs. High, C = Low vs. High

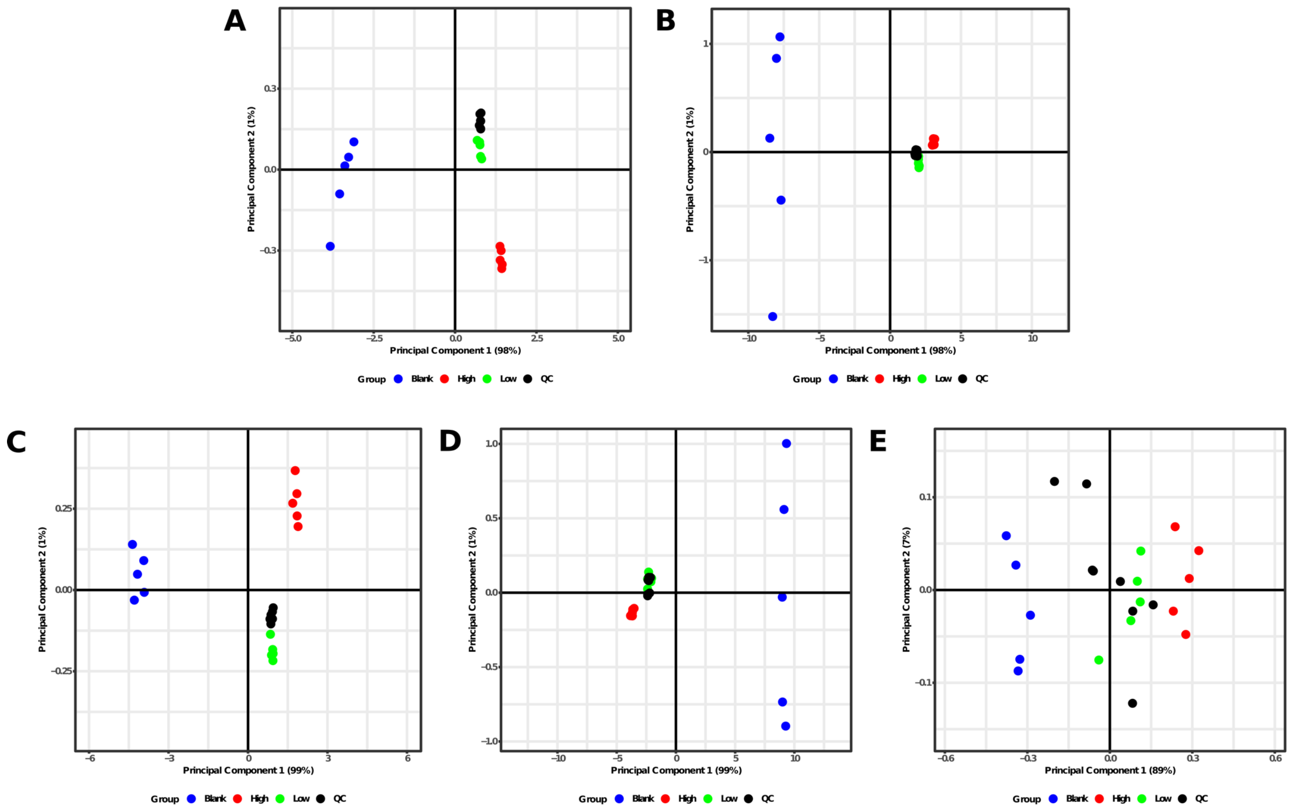

Figure S9. Scores of principal component analysis. A = alpha-PBP, PhenylHexyl column, positive mode; B = alpha-PBP, HILIC column, positive mode; C = alpha-PEP, PhenylHexyl column, positive mode; D = alpha-PEP, HILIC column, positive mode; E = alpha-PEP, HILIC column, negative mode.

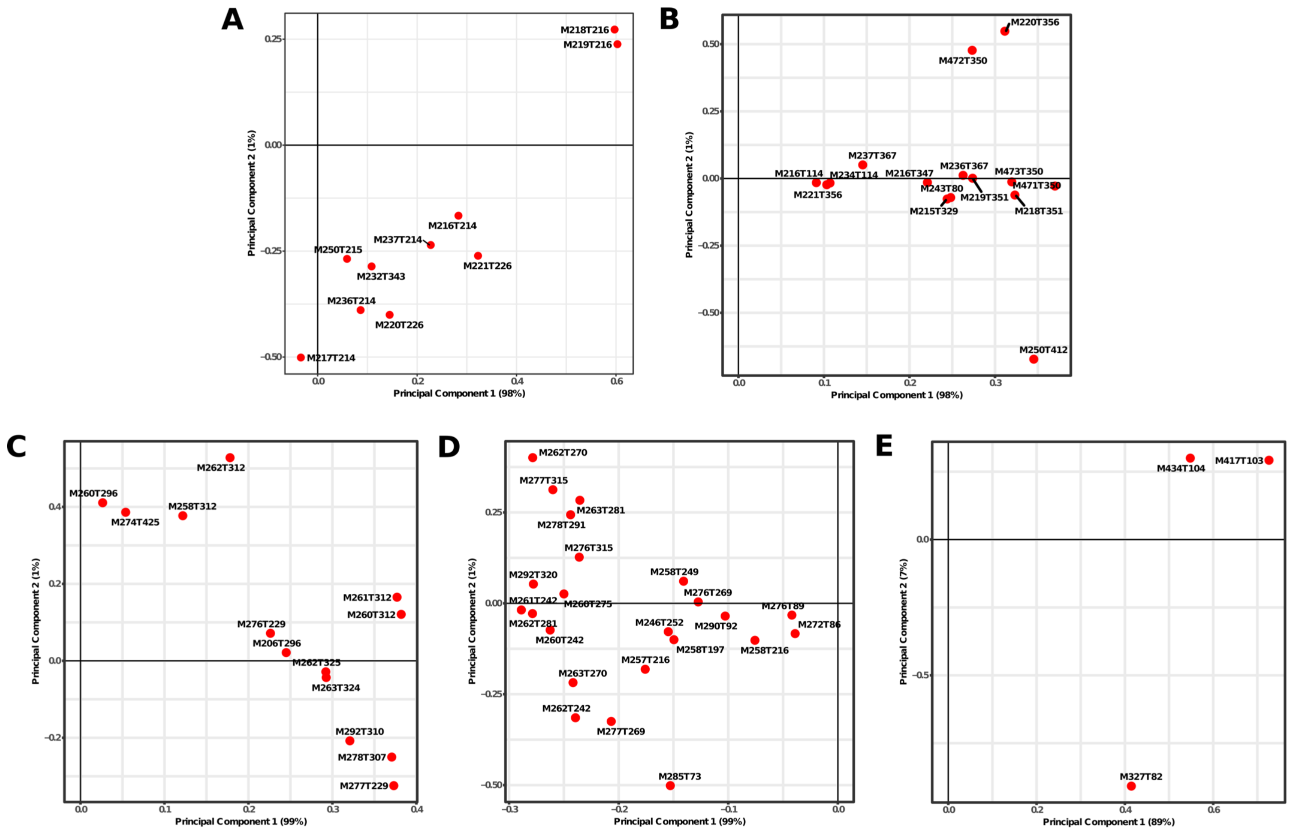

Figure S10. Loadings of principal component analysis. A = alpha-PBP, PhenylHexyl column, positive mode; B = alpha-PBP, HILIC column, positive mode; C = alpha-PEP, PhenylHexyl column, positive mode; D = alpha-PEP, HILIC column, positive mode; E = alpha-PEP, HILIC column, negative mode.

M218T216, alpha-PBP

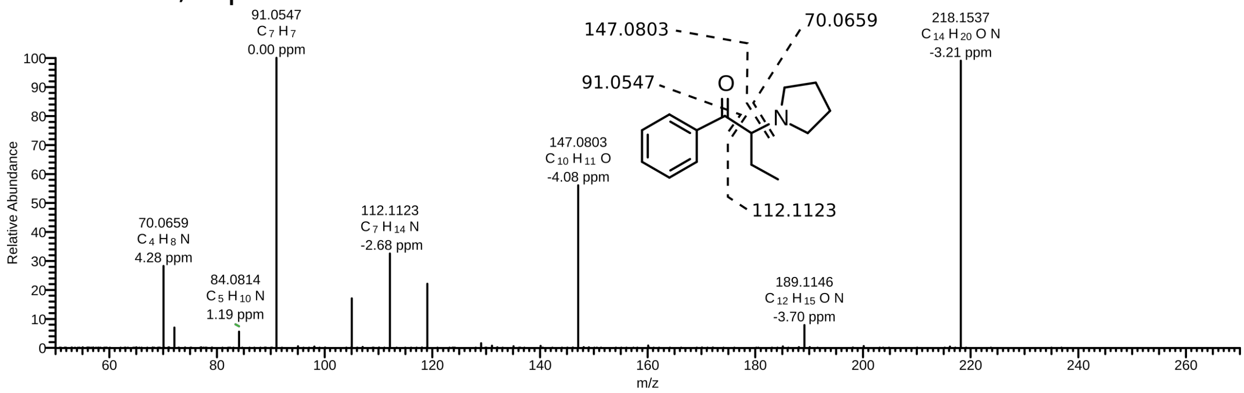

M216T214, alpha-PBP artifact (dehydro-)

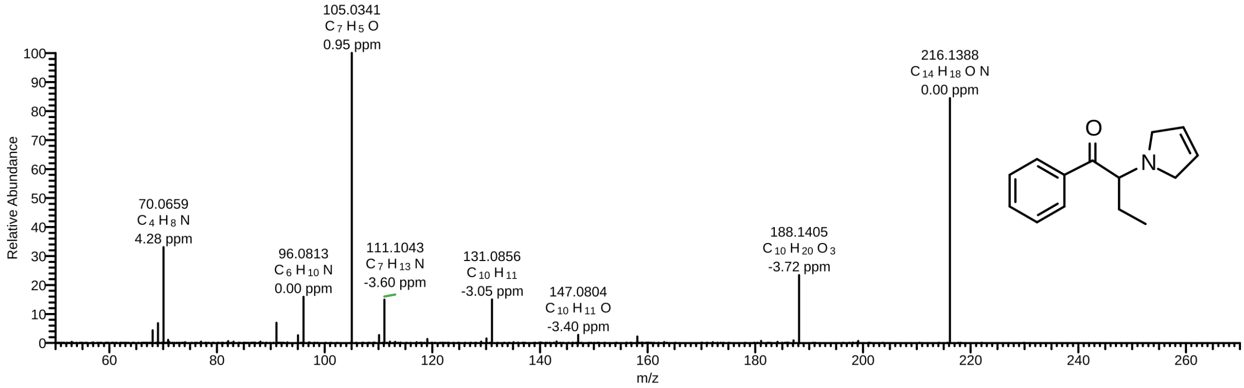

M220T226, alpha-PBP-M (dihydro-)

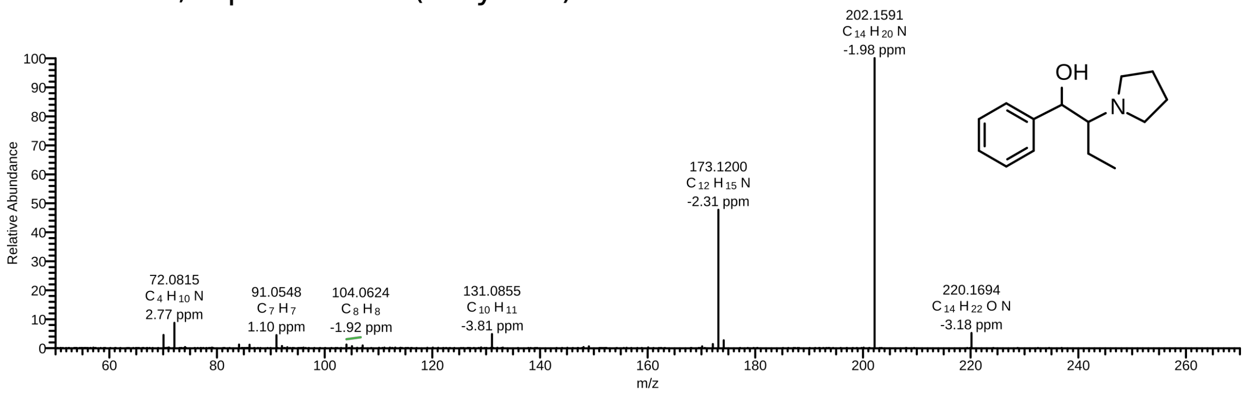

M232T343, alpha-PBP-M (oxo-)

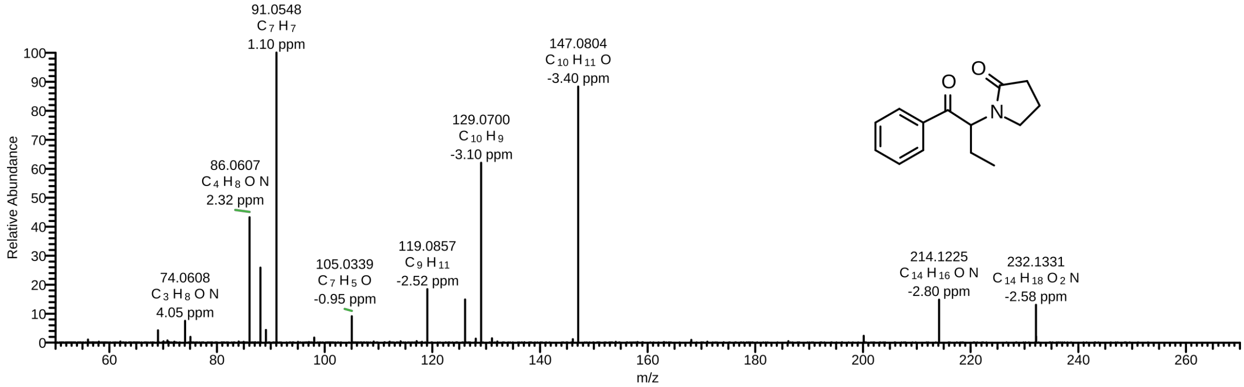

Figure S11. LC-HR-MS/MS spectra of significant features after incubation with alpha-PBP using a PhenylHexyl column and positive ionization mode. Fragments with accurate mass, calculated elemental formula, and mass error value in parts per million (ppm).

M236T214, alpha-PBP-M (dihydro-oxo-)

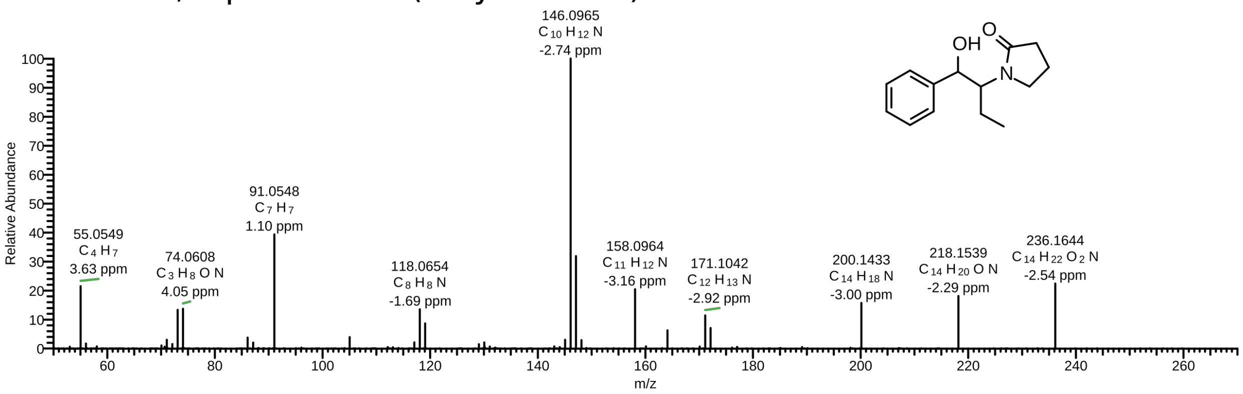

M250T215, alpha-PBP-M (di-HO-)

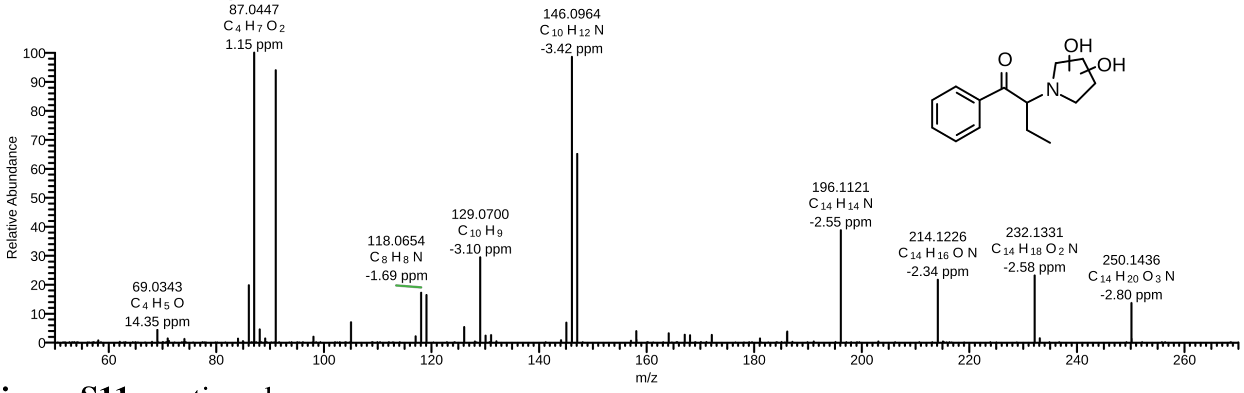

Figure S11. continued.

M218T351, alpha-PBP

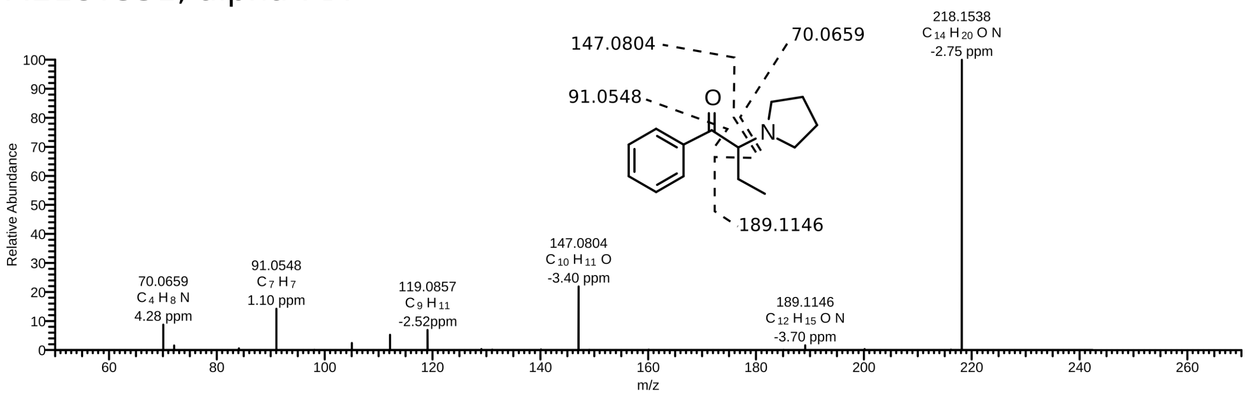

M215T328, alpha-PBP impurity (dehydro-) artifact (imido-)

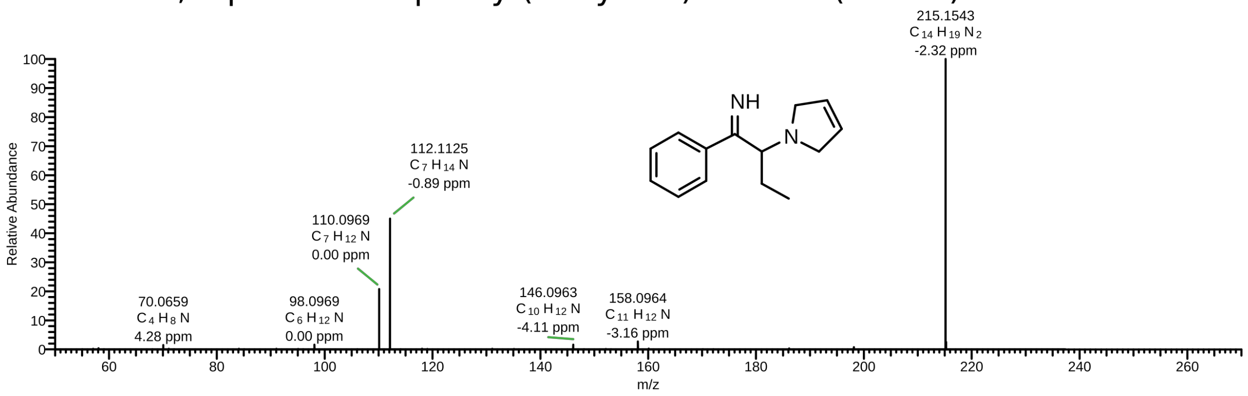

M216T114, alpha-PBP-M (dihydro-oxo-) artifact

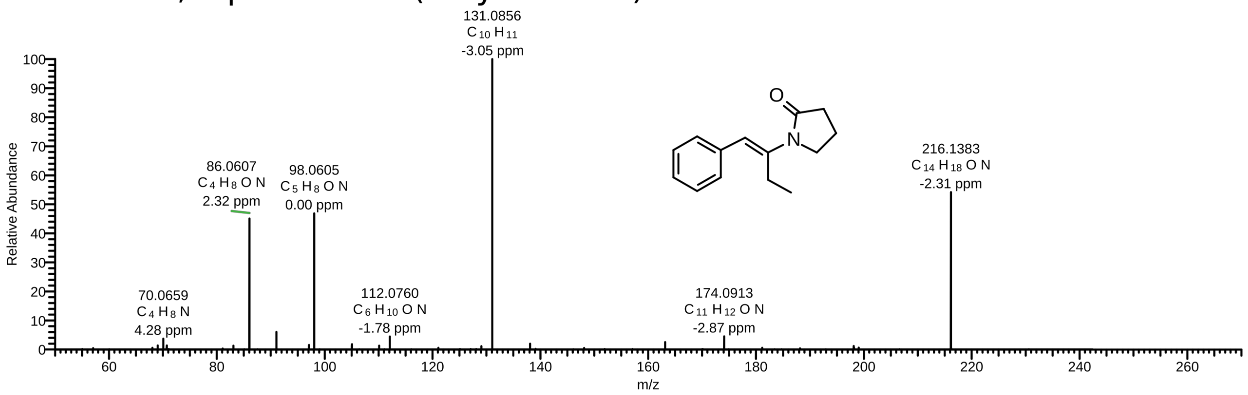

M216T347, alpha-PBP artifact (dehydro-)

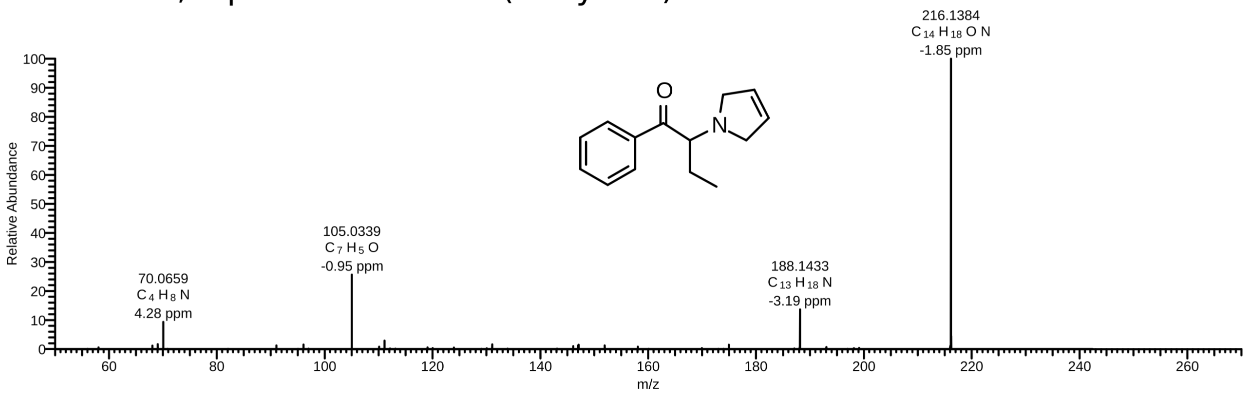

Figure S12. LC-HR-MS/MS spectra of significant features after incubation with alpha-PBP using a HILIC column and positive ionization mode. Fragments with accurate mass, calculated elemental formula, and mass error value in parts per million (ppm).

M220T355, alpha-PBP-M (dihydro-)

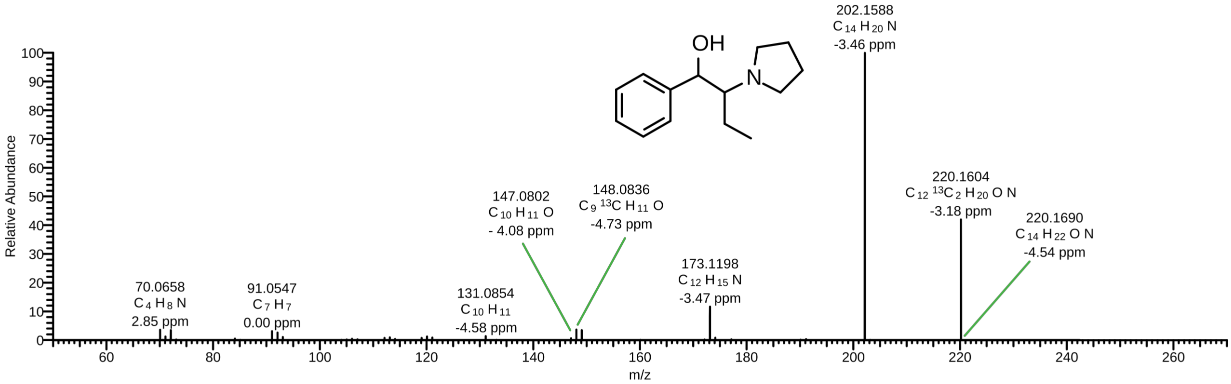

M234T114, alpha-PBP-M (dihydro-oxo-)

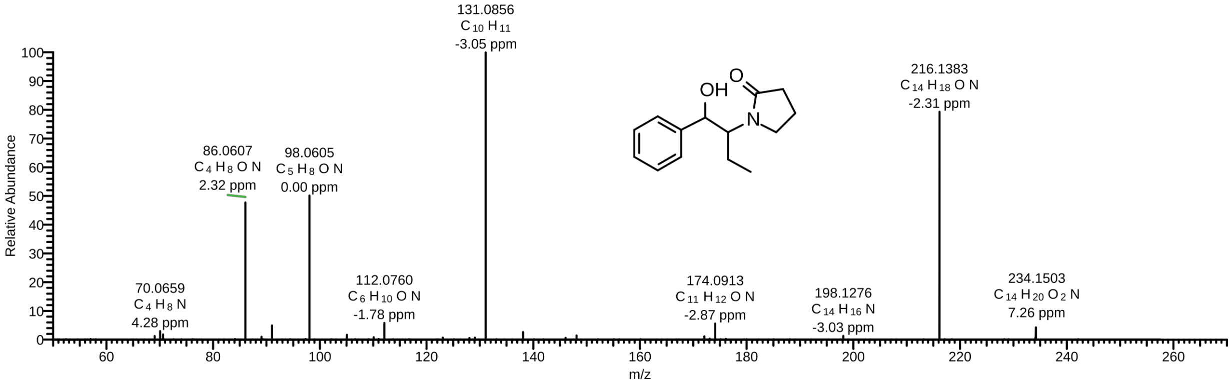

M236T367, alpha-PBP-M (dihydro-HO-)

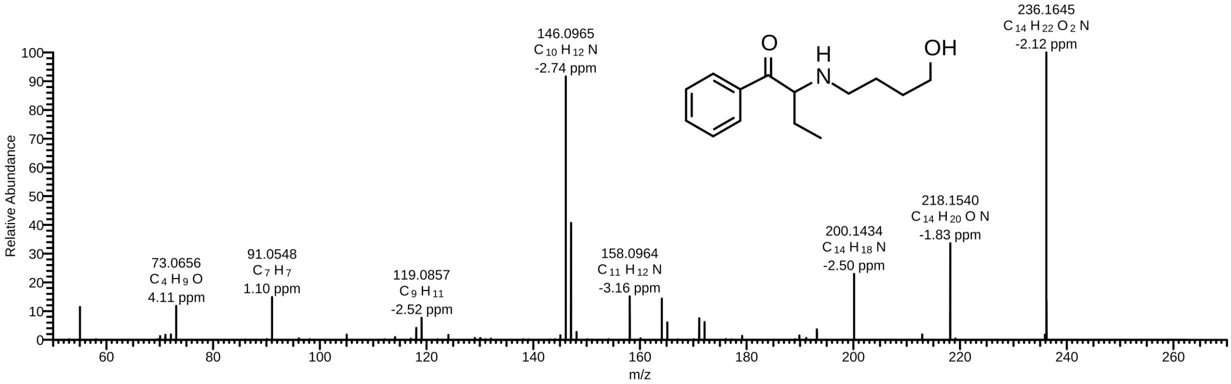

M243T80, alpha-PBP impurity (dehydro-) artifact (cyano-)

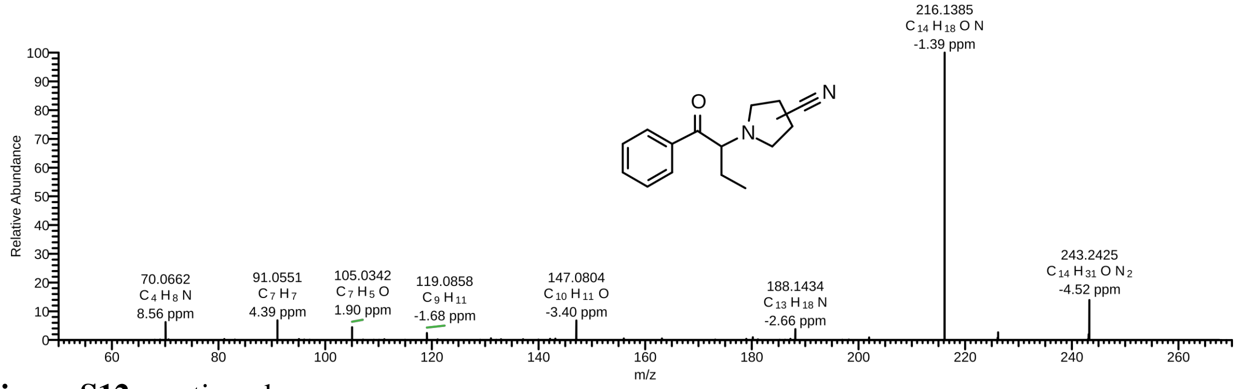

Figure S12. continued.

M250T412, alpha-PBP-M (di-HO-)

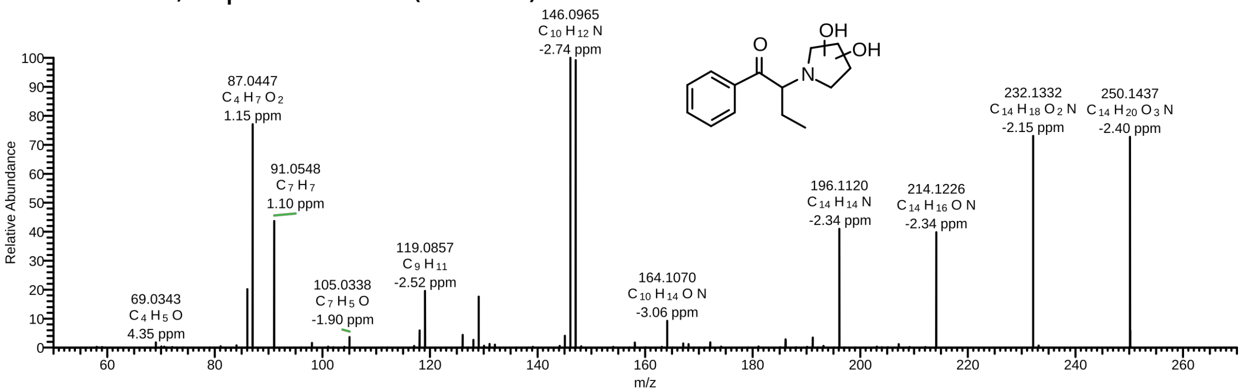

M471T350, alpha-PBP adduct [2M+2H+Cl]+

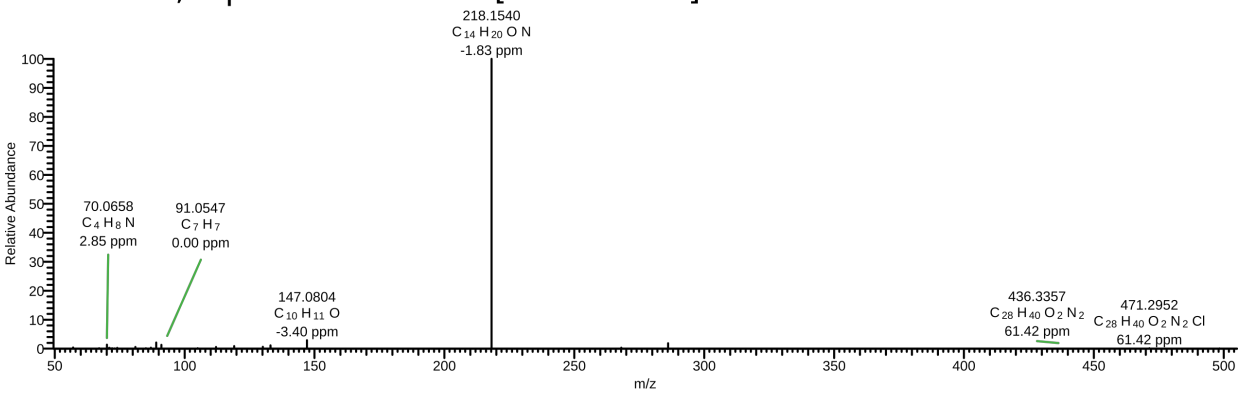

Figure S12. continued.

M260T312, alpha-PEP conformer 1

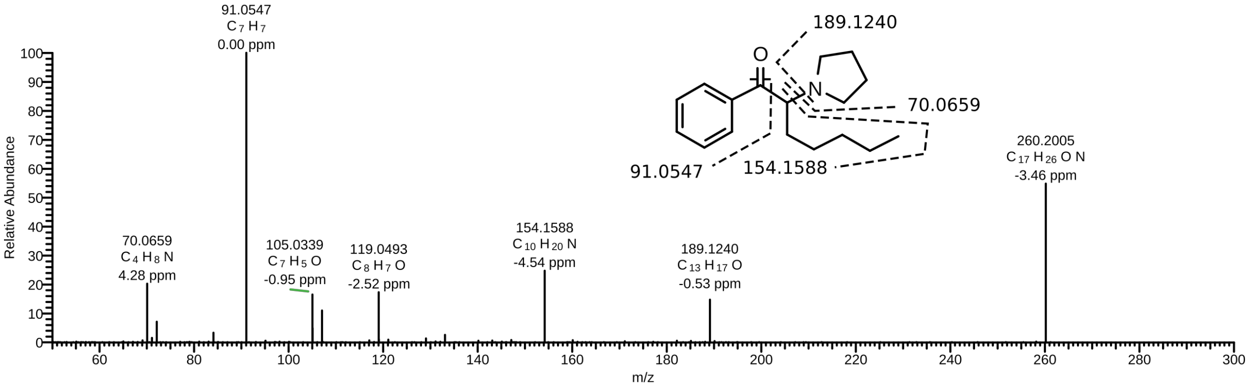

M260T296, alpha-PEP conformer 2

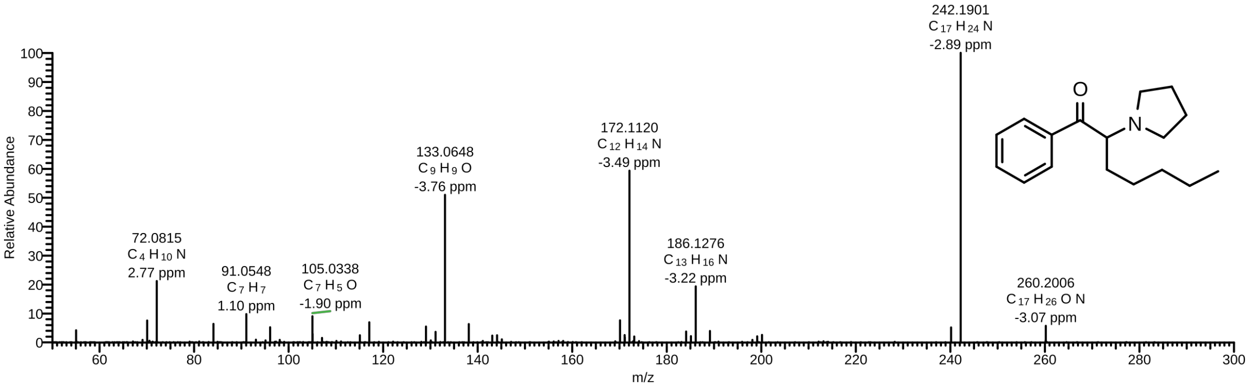

M206T296, alpha-PEP-M (*N*,*N*-dealkyl-)

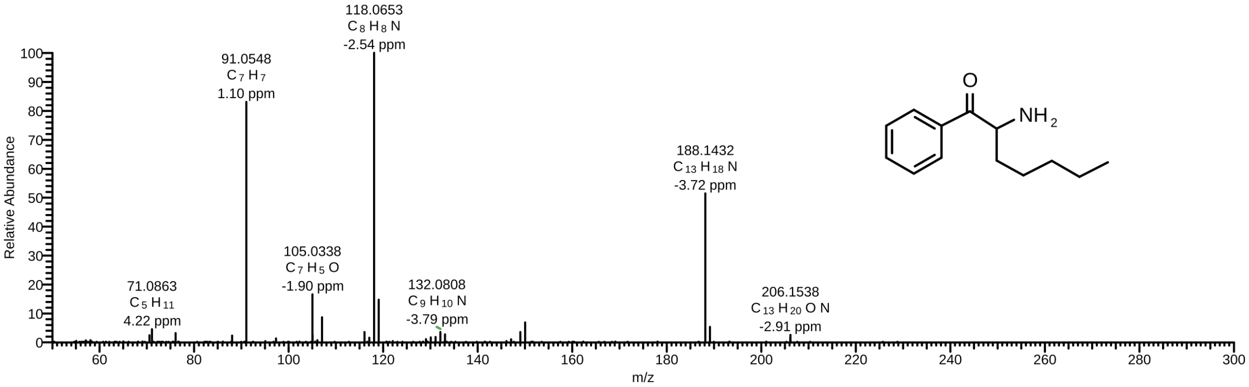

M258T312, alpha-PEP artifact (dehydro-)

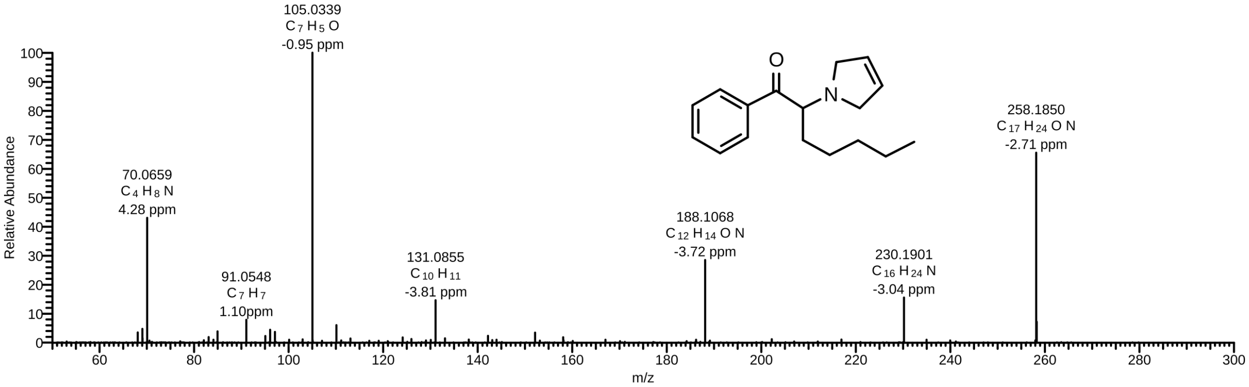

Figure S13. LC-HR-MS/MS spectra of significant features after incubation with alpha-PEP using a PhenylHexyl column and positive ionization mode. Fragments with accurate mass, calculated elemental formula, and mass error value in parts per million (ppm).

M262T325, alpha-PEP-M (dihydro-)

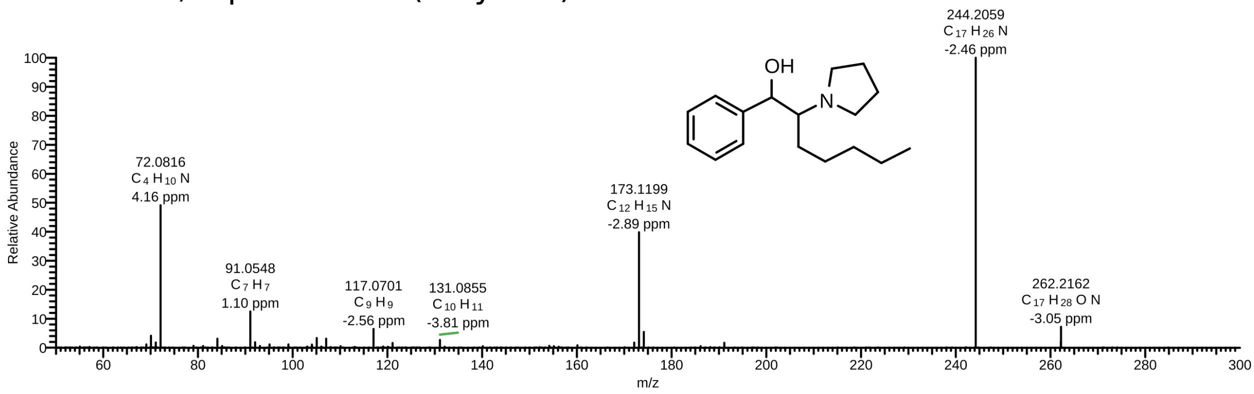

M274T425, alpha-PEP-M (oxo-)

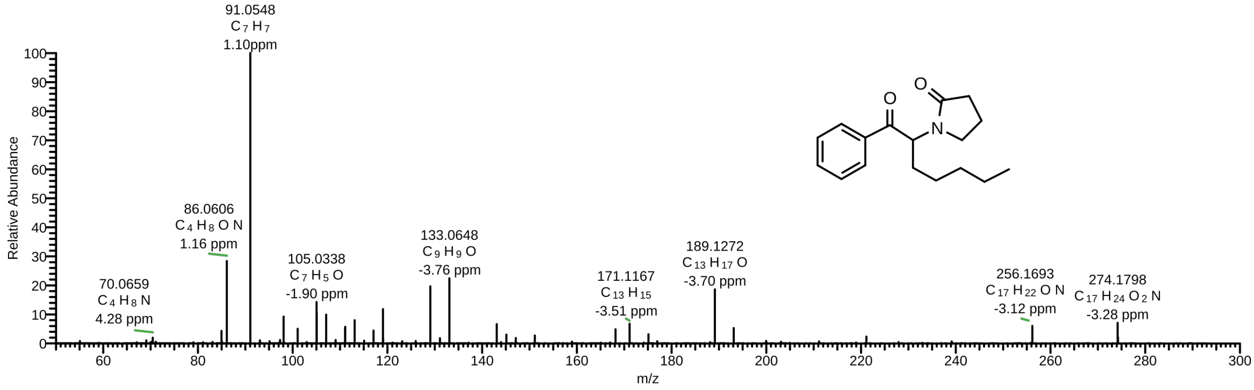

M276T307, alpha-PEP-M (HO-) isomer 2

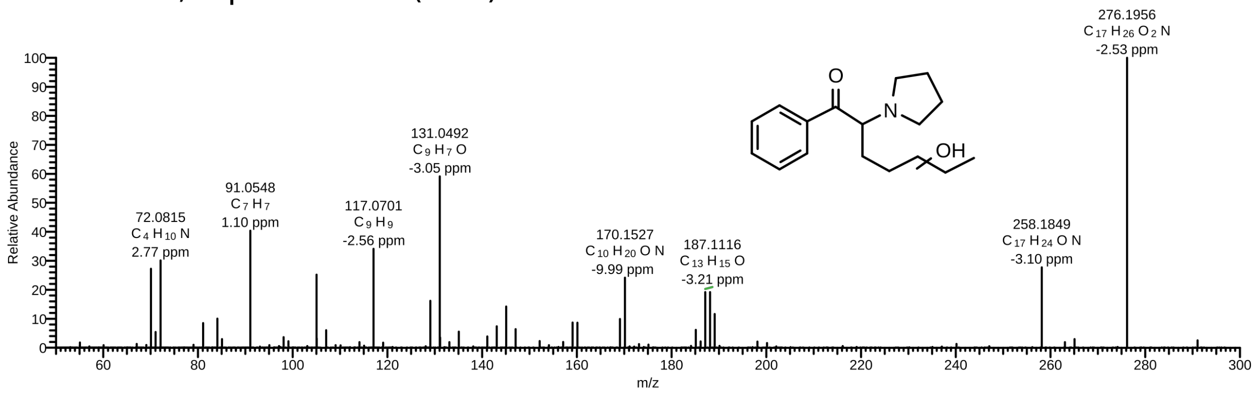

M278T307, alpha-PEP-M (dihydro-HO-)

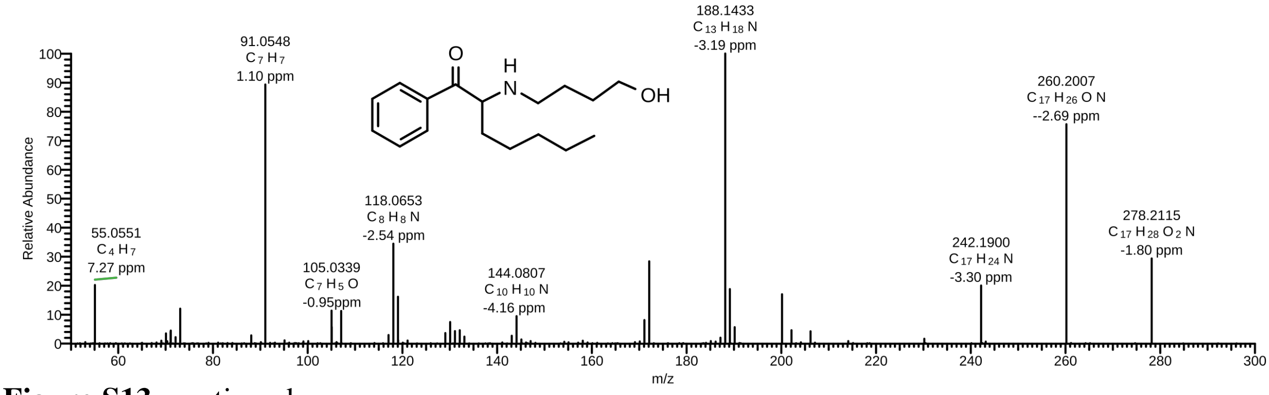

Figure S13. continued.

M292T310, alpha-PEP-M (di-HO-)

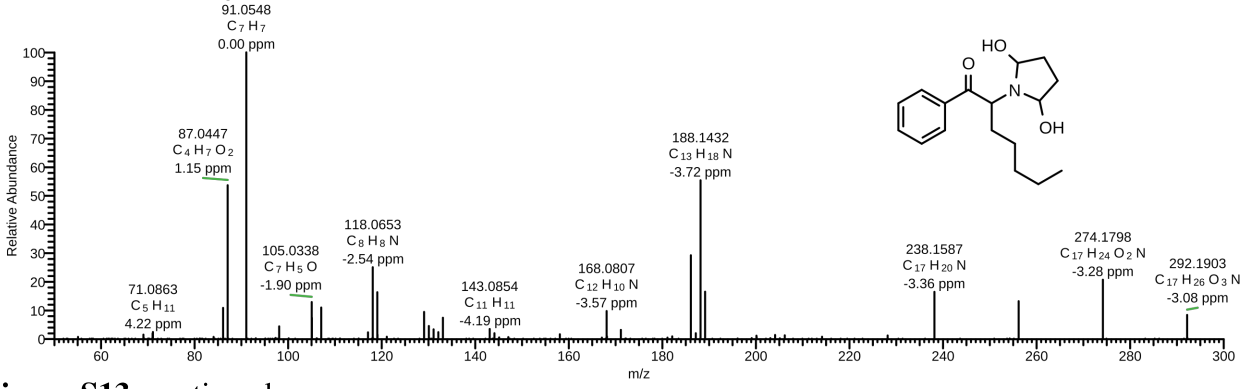

Figure S13. continued.

M260T243, alpha-PEP conformer 1

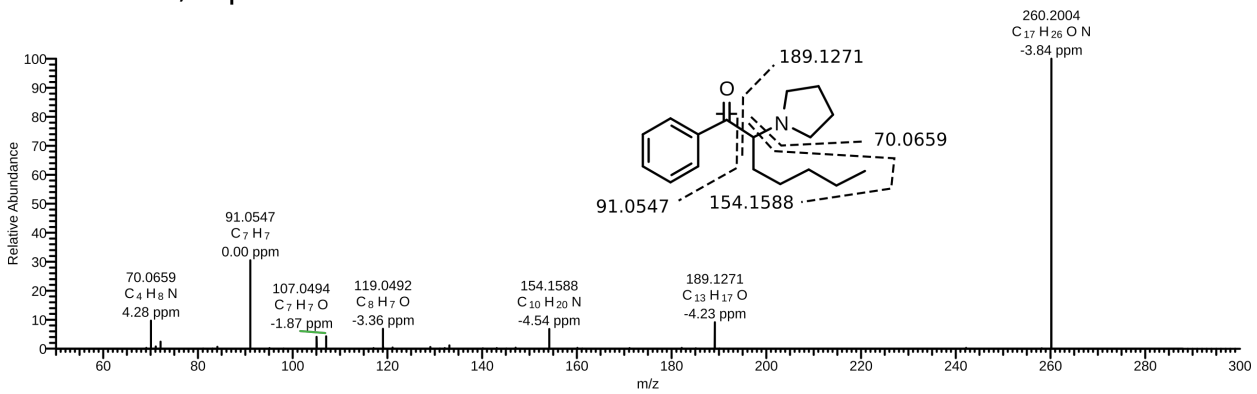

M260T276, alpha-PEP conformer 2

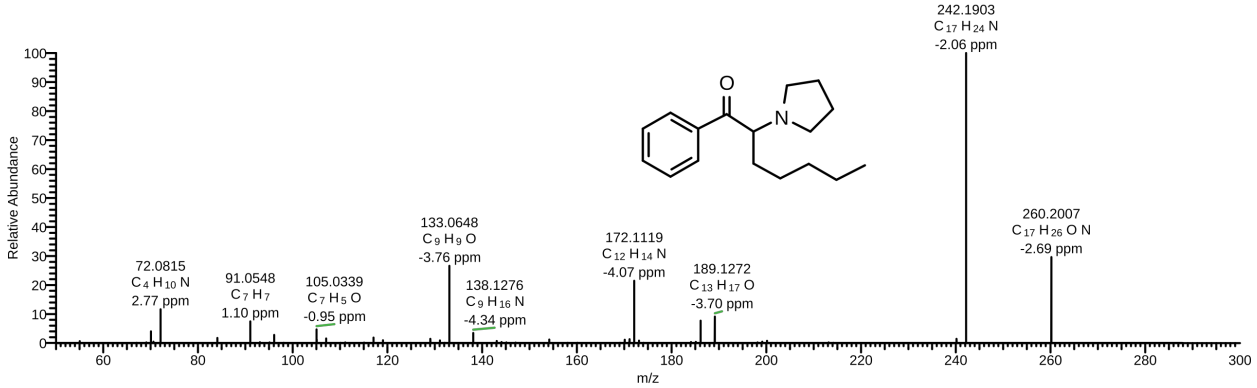

M246T254, alpha-PEP impurity (alpha-PHP)

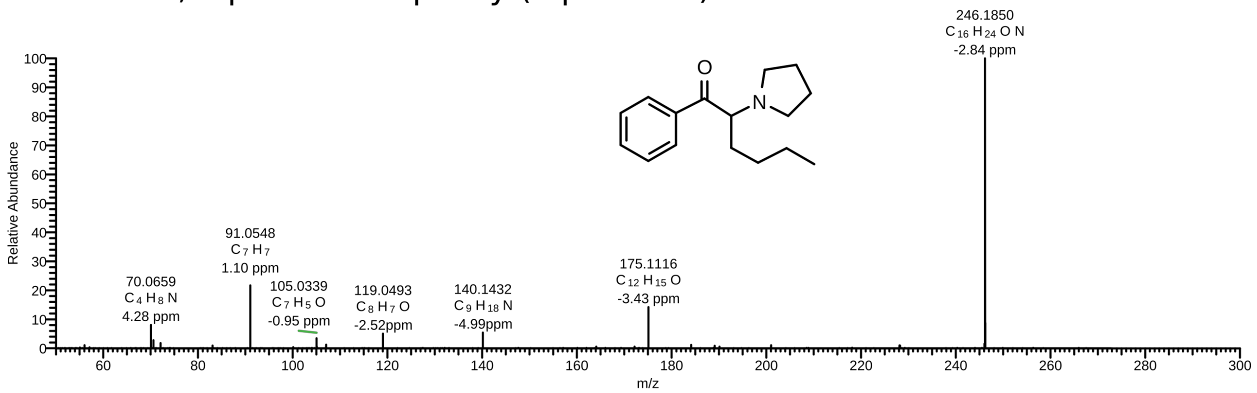

M257T216, alpha-PEP impurity (dehydro-) artifact (imido-)

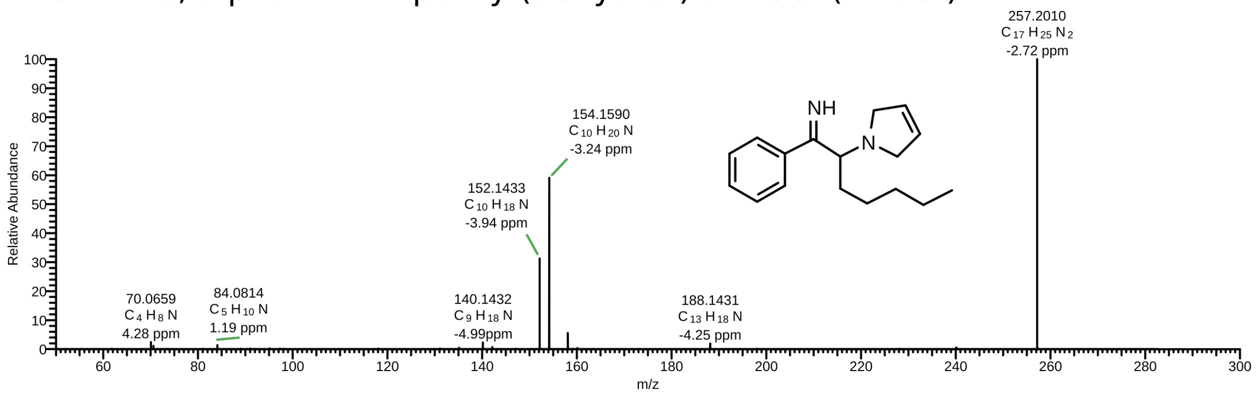

Figure S14. LC-HR-MS/MS spectra of significant features after incubation with alpha-PEP using a HILIC column and positive ionization mode. Fragments with accurate mass, calculated elemental formula, and mass error value in parts per million (ppm).

M258T198, alpha-PEP impurity (dehydro-)

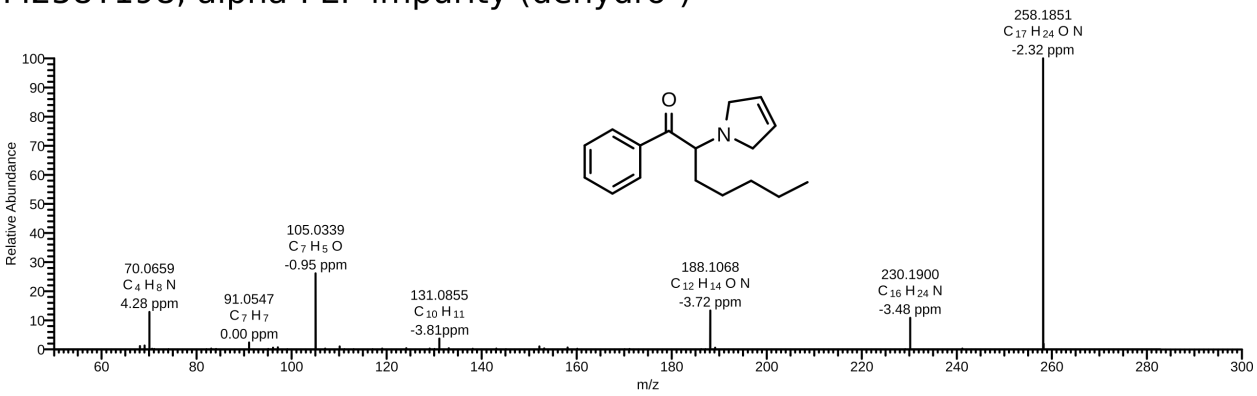

M258T249, alpha-PEP artifact (dehydro-)

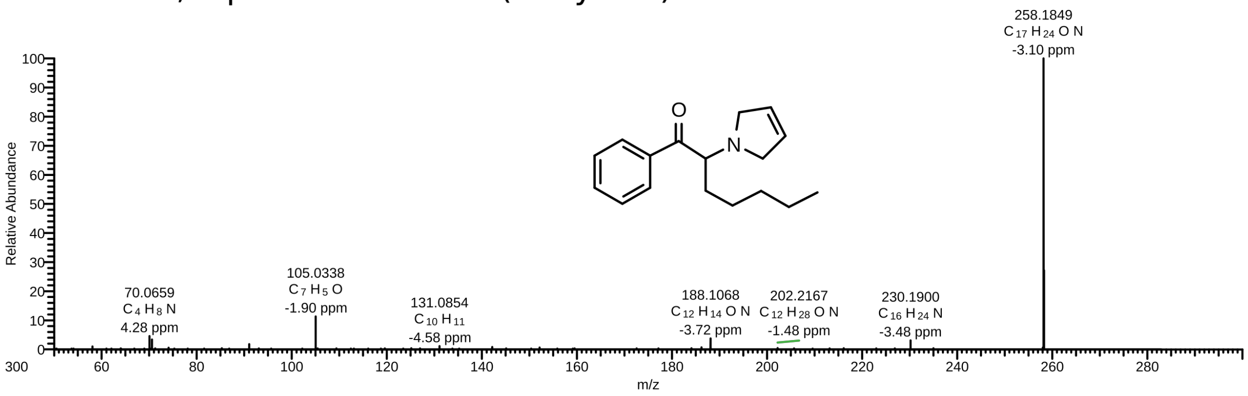

M262T271, alpha-PEP-M (dihydro-) diastereomer 1

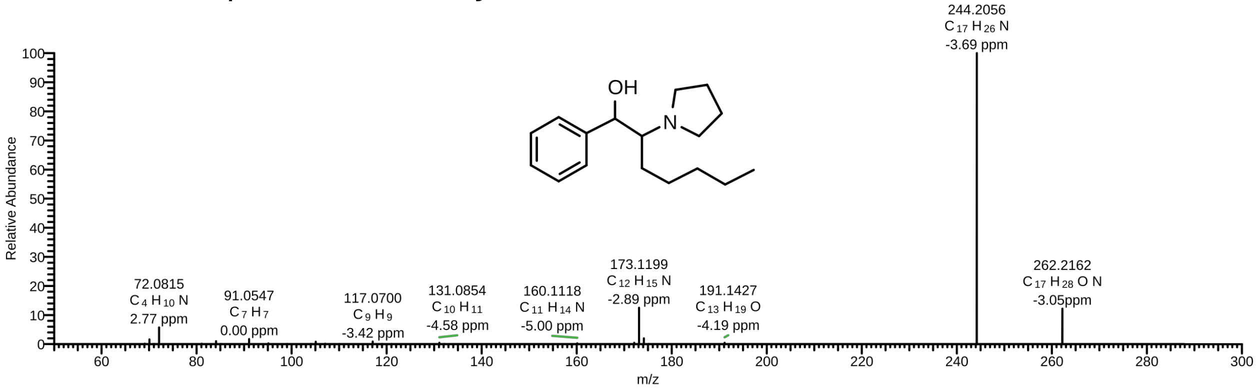

M262T282, alpha-PEP-M (dihydro-) diastereomer 2

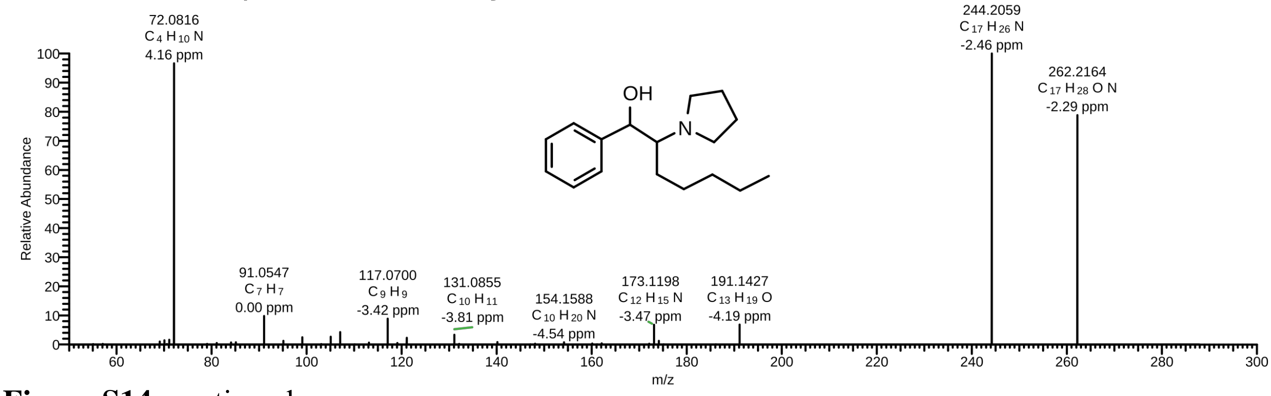

Figure S14. continued.

M272T87, alpha-PEP-M (oxo-HO-) isomer 2 artifact (dehydro-)

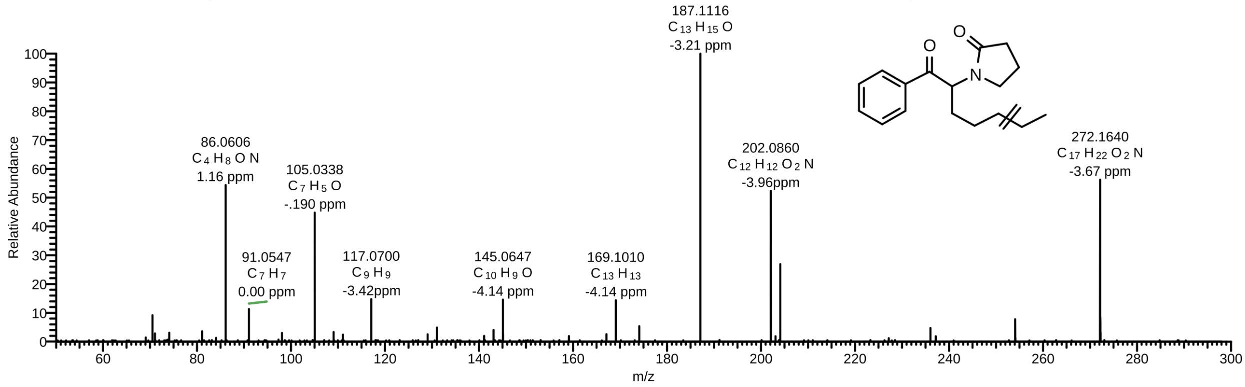

M276T90, alpha-PEP-M (dihydro-oxo-)

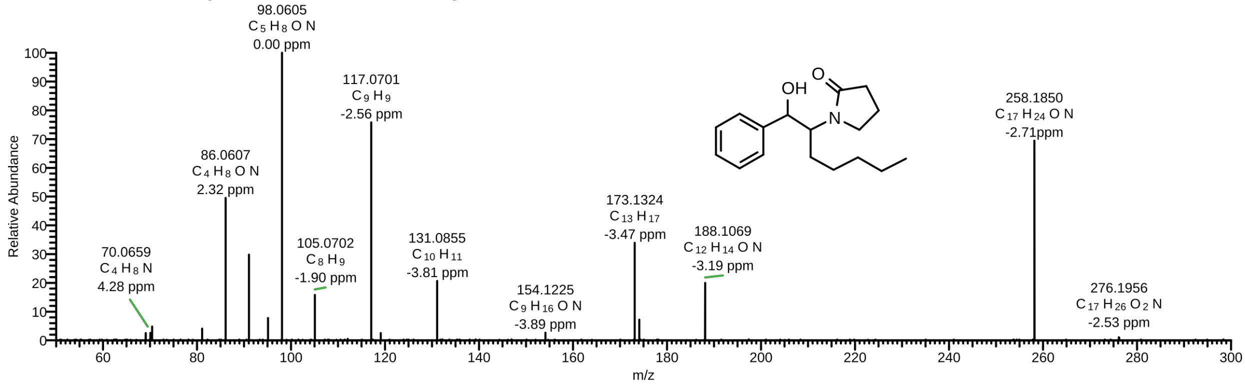

M276T229, alpha-PEP-M (HO-) isomer 2

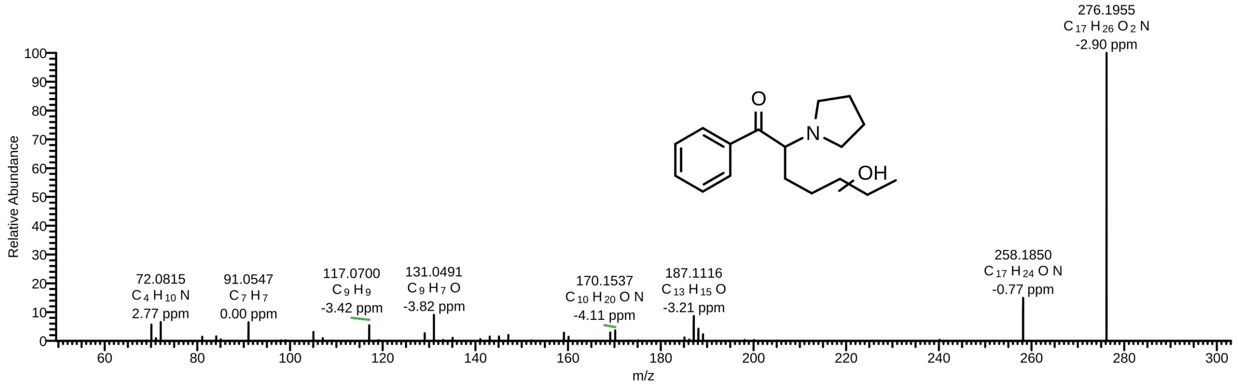

M279T269, alpha-PEP-M (HO-) isomer 1

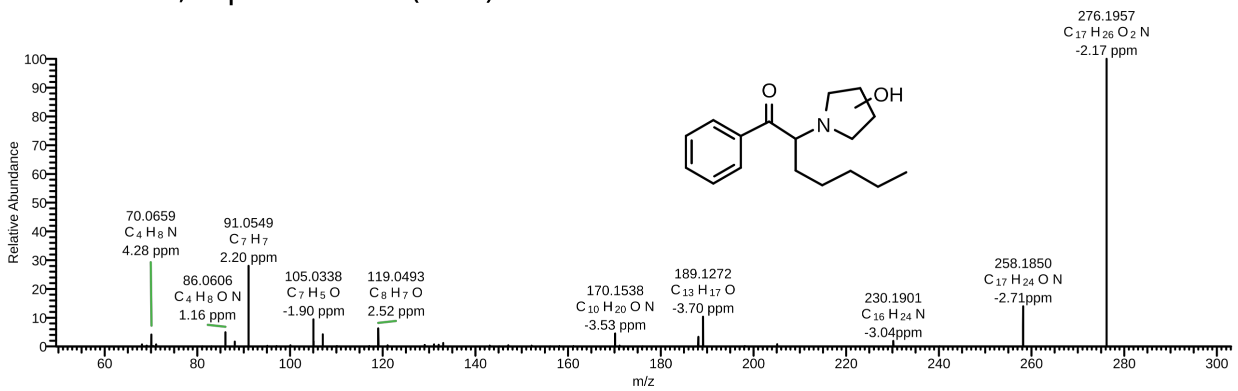

Figure S14. continued.

M278T291, alpha-PEP-M (dihydro-HO-)

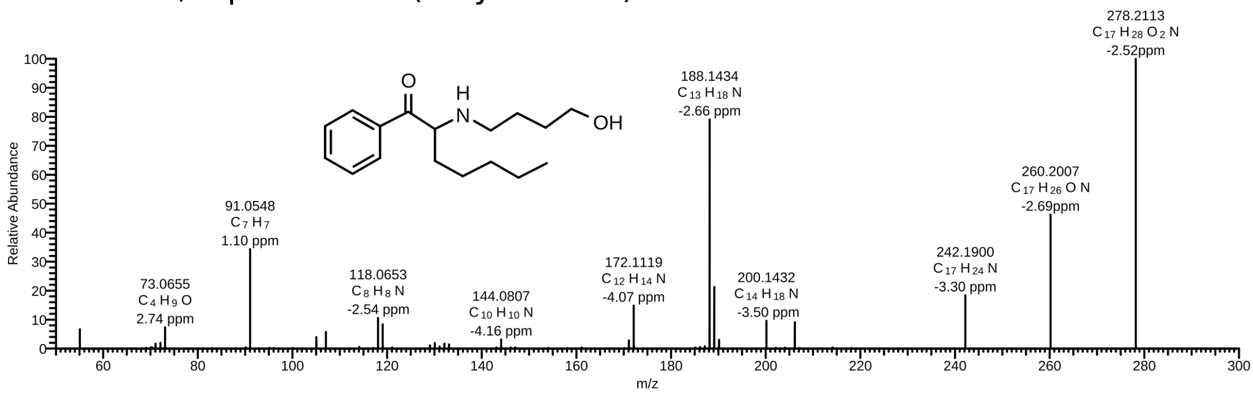

M285T74, alpha-PEP artifact (cyano-)

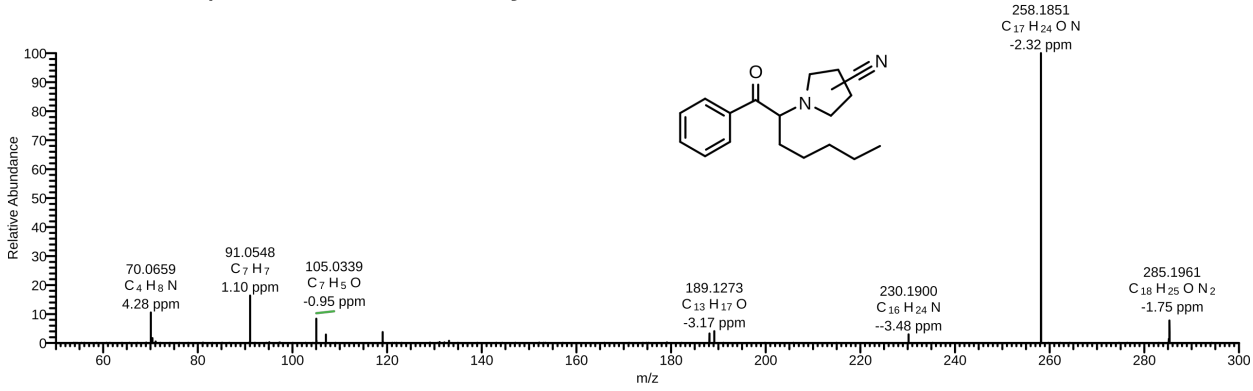

M290T93, alpha-PEP-M (oxo-HO-) isomer 1

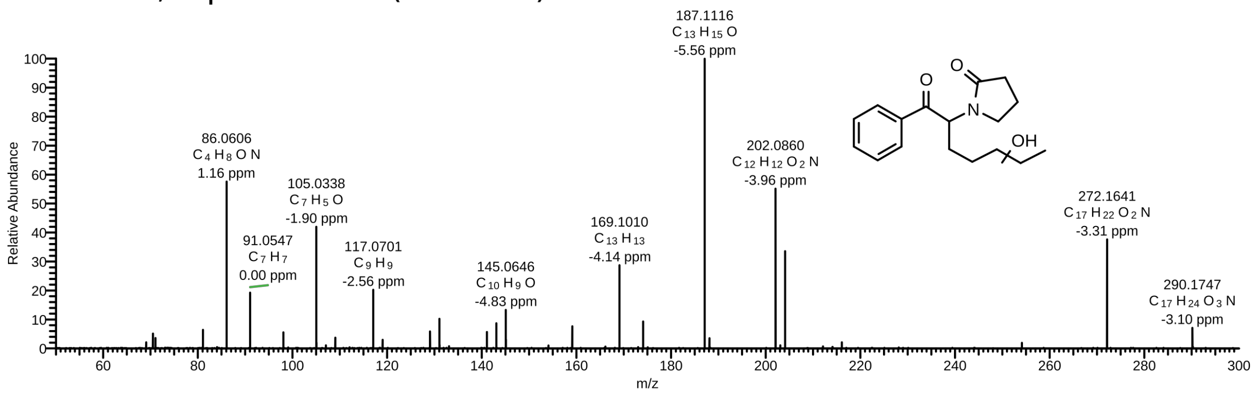

M292T321, alpha-PEP-M (di-HO-)

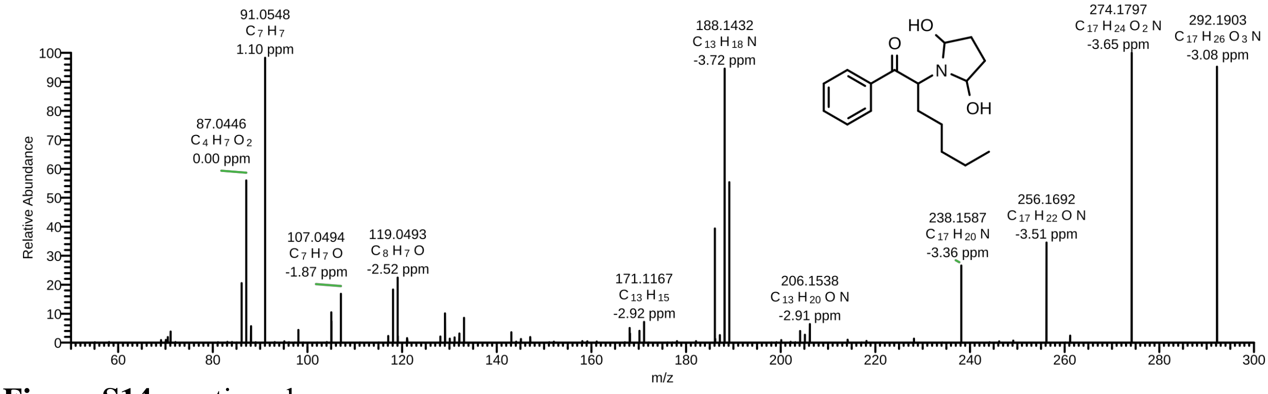

Figure S14. continued.

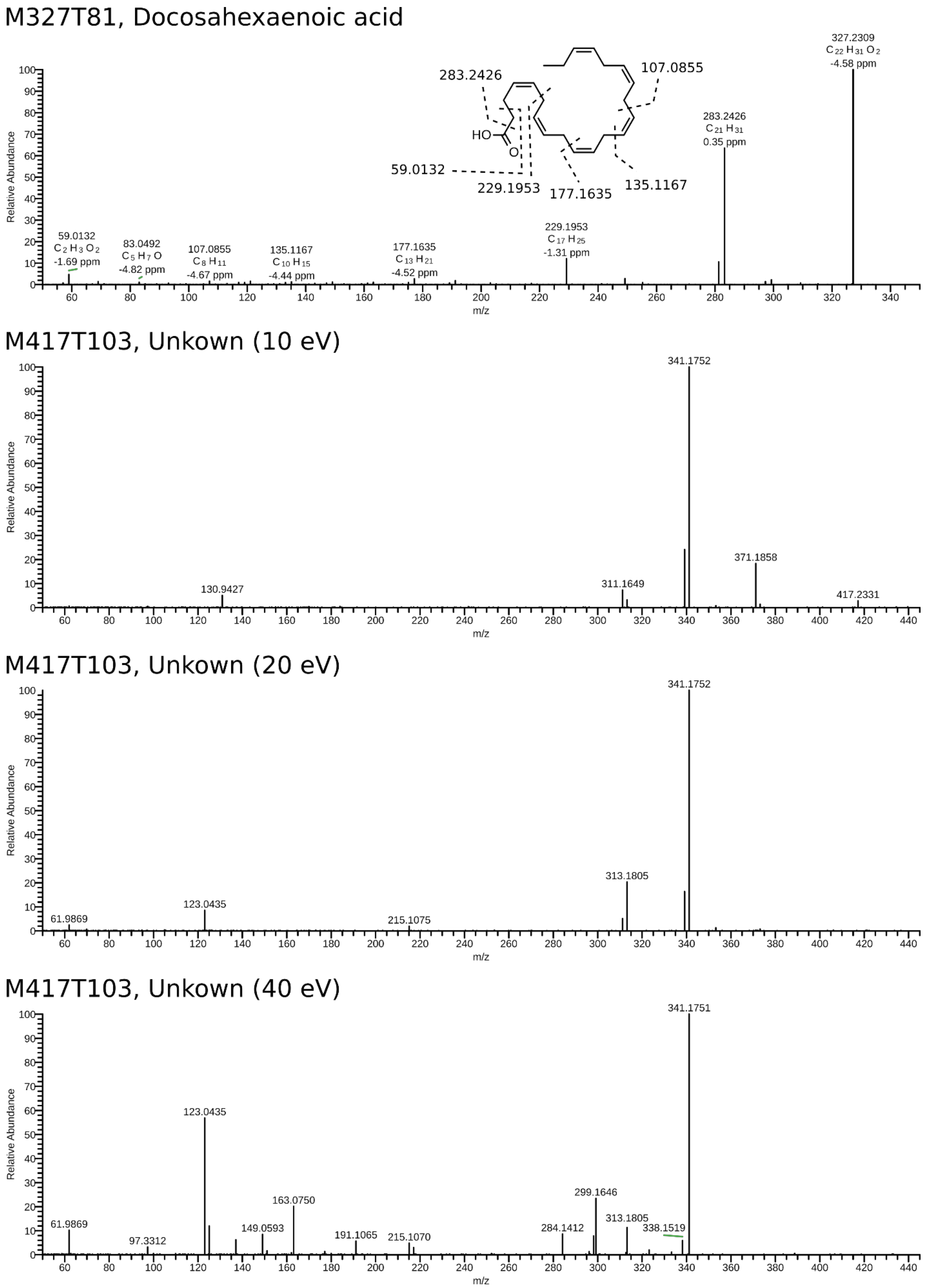

Figure S15. LC-HR-MS/MS spectra of significant features after incubation with alpha-PEP using a HILIC column and negative ionization mode. Fragments with accurate mass, as well as calculated elemental formula and mass error value in parts per million (ppm) for identified compounds.

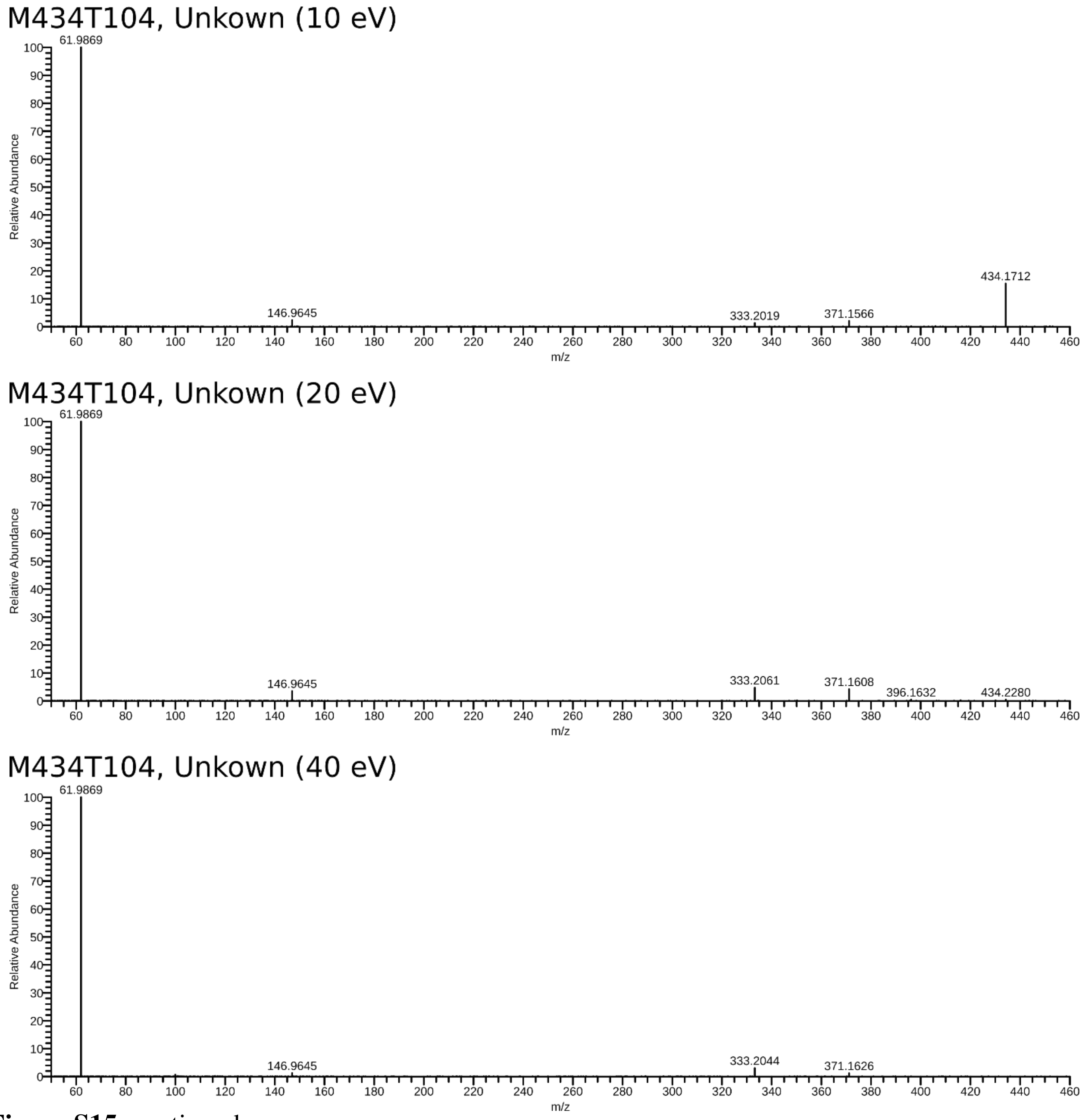

Figure S15. continued.

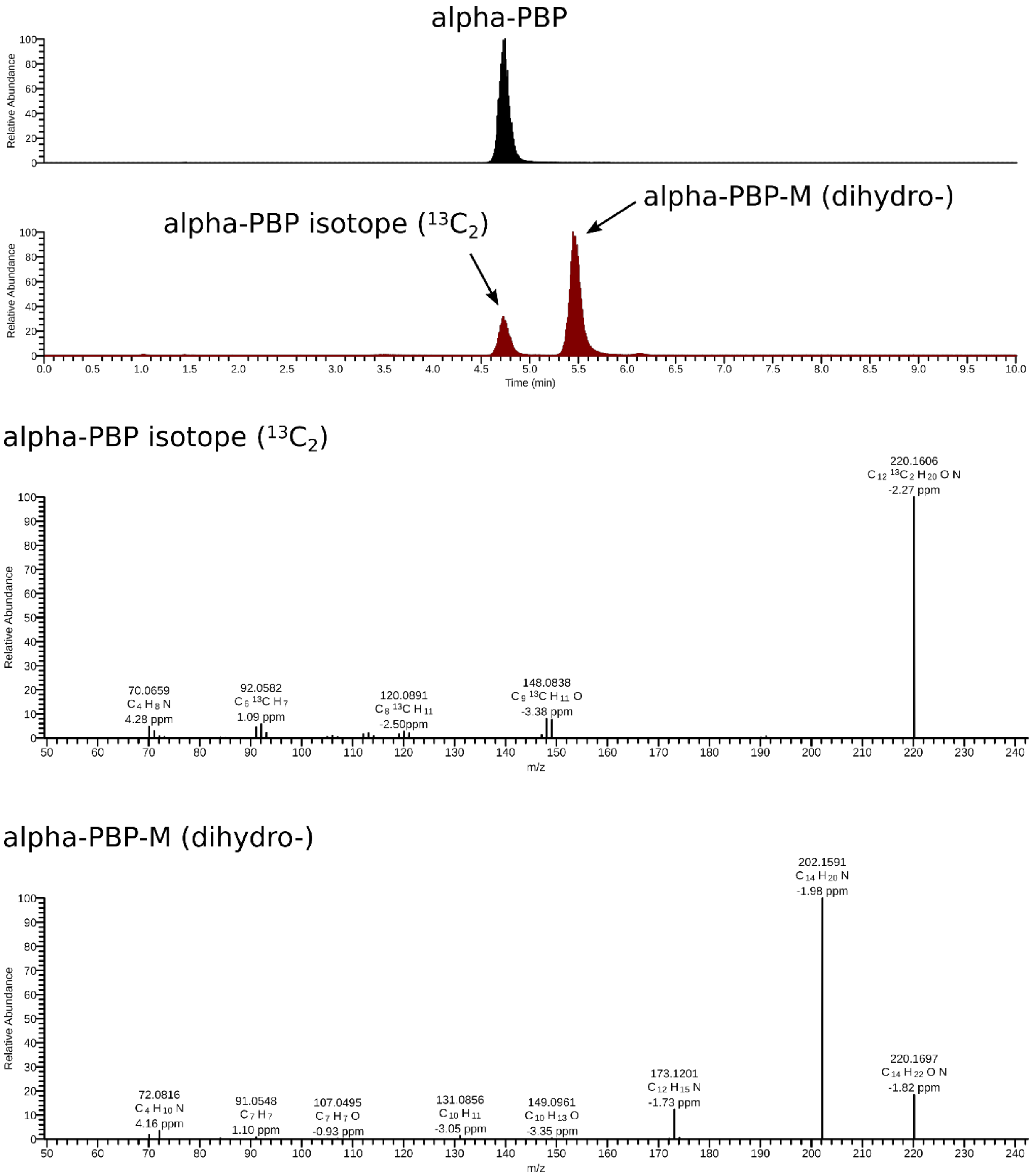

Figure S16. Separation of alpha-PBP isotope ($^{13}C_2$) and alpha-PBP-M (dihydro-) after analysis using an alternative chromatography. The following gradient was used: 0-1.0 min 2% C, 1-10 min to 15% C, 8.5-10 min hold 60% C. 10-12 min hold 2%.
